# Educational trajectories within and beyond the core education phase in Switzerland: a sequence analysis based on SHP data 1999–2023

**DOI:** 10.3389/fsoc.2025.1585910

**Published:** 2025-06-05

**Authors:** Susanne Edler, Andreas Hadjar

**Affiliations:** Division Sociology, Social Policy, Social Work, Université de Fribourg, Fribourg, Switzerland

**Keywords:** educational trajectories, educational inequalities, educational expansion, sequence analysis, Switzerland

## Abstract

**Introduction:**

Switzerland's educational expansion has lagged behind other industrialized countries, with fewer youths attending upper secondary schools and attaining tertiary education. This paper examines individual educational trajectories over a long period, considering later achievements and higher vocational qualifications. It also identifies inequalities in educational trajectory patterns based on social and migration background, as well as gender.

**Methods:**

Using Swiss Household Panel (SHP) data from 1999 to 2023, the sample includes 2,580 individuals born between 1939 and 1983.

**Results:**

Findings show an extended education phase over time, with increased higher education and vocational training. Fewer individuals completed their education by age 21 in the 1969–83 cohort compared to earlier cohorts. Three clusters were identified for the 1939–53 cohort, six for the 1954–68 cohort, and three again for the 1969–83 cohort, indicating diversification.

**Discussion:**

Educational trajectory patterns vary significantly among different social groups, highlighting profound inequalities.

## 1 Introduction

Switzerland is often characterized as a country that has experienced a slower educational expansion compared to other Western and Eastern industrialized countries, with relatively low percentages of the youth population attending upper secondary schools and attaining tertiary education (Becker and Zangger, 2013; Becker and Jann, 2017; Buchmann et al., 1993, 2016; Schüpbach, 2014). Educational attainment is typically measured as an immediate outcome following compulsory education, assuming ideal transition sequences within the education system. However, later educational achievements and tertiary vocational qualifications are less frequently considered. From this perspective, contributing to research on educational attainment and educational inequality by considering alternative educational pathways—such as further education or enrolling in evening courses for working adults—and studying individual educational and work trajectories over a longer period appears to be promising.

On one hand, research suggests that in the stratified cantonal education systems in Switzerland (Hadjar and Uusitalo, 2016) educational underachievement is largely irreversible and socially inherited, with those failing to achieve educational milestones in youth unlikely to do so later (Schräder-Naef, 1997; Schräder-Naef and Jörg-Fromm, 2005). On the other hand, Meyer (2018) demonstrates that, in Switzerland, transitions between education and employment extend into the third decade of life and beyond, characterized by reorientations, interruptions, and interim periods. Notably, around 40% of young people continue their education at the tertiary level, often after a period of employment rather than immediately following secondary education (Meyer, 2018).

Against this backdrop, this paper focuses on three research questions: (1) Which educational trajectory patterns can be identified in the stratified Swiss education system? (2) How do educational trajectory patterns change during educational expansion, and thus over successive birth cohorts? and (3) Can inequalities related to social origin, gender, and migration background be found in these typical educational trajectory patterns? Studying these research questions, we adopt a longitudinal perspective spanning 25 years to offer a more nuanced depiction of the complexity of educational trajectories. Using Swiss Household Panel (SHP) data from 1999 to 2023, a sequence analysis is conducted to examine patterns of educational trajectories throughout the life course in Switzerland. Multinomial logistic regression models are employed to assess inequalities in educational trajectory patterns.

First, we will introduce the life course perspective as a major paradigm for the study of trajectories, contrasting this approach with single transitions or educational stages, before theorizing educational expansion as the main background process behind the expected cohort differences in educational trajectories. Second, we will contextualize the Swiss case and describe typical characteristics of the cantonal education systems in Switzerland in detail. Third, we will transparently describe the methodology including data set, operationalizations, and analytical procedures. We will then present the results of sequence analysis, cluster analysis and multinomial logistic regression models, before finally discussing our findings in relation to the research questions and the broader state-of-research.

## 2 Conceptual considerations

### 2.1 The life course approach in educational research

The development of educational and career paths extends beyond the completion of compulsory schooling. Our study applies a life course perspective to explore how these trajectories evolve over time, emphasizing the importance of understanding social mobility and educational attainment through the lens of individuals' entire life. The life course approach suggests integrating various stages and transitions in life, such as family background, education, and employment, to provide a comprehensive view of social inequality and mobility (Hillmert and Jacob, 2005, 2010; Hillmert, 2015). This perspective is particularly meaningful in this context, as educational and career pathways are often complex, non-linear and involve multiple detours or transitions (Hillmert and Jacob, 2005; Hillmert, 2015). By accounting for these complexities, the life course approach provides a more accurate representation of individuals' experiences. Additionally, by examining the impact of social background on educational and career outcomes, the life course approach sheds light on the mechanisms of intergenerational mobility and reproduction of social inequality. Looking beyond the core phase of education toward later educational outcomes allows for a better understanding of educational participation and inequalities. Early educational decisions, failures, and low aspirations—to some extent shaped by inequalities between different social groups (Hillmert and Jacob, 2005)—may be compensated for later in life. The timing of key life events, such as entering the labor market or returning to education, plays a crucial role in shaping individuals' life trajectories. As noted by Elder (1998, p. 3) (one of the pioneers of life course research): “the developmental impact of a succession of life transitions or events is contingent on when they occur in a person's life”. While this quote relates to changes during individual trajectories (personal development and aging), the life course approach also takes into account how individual trajectories are shaped by external processes contingent upon historical times and events (Elder, 1999). When significant societal changes differentiate the lives of cohorts, a lifelong cohort effect is created amongst members of a birth cohort who all share a distinctive formative experience (Alwin and McCammon, 2003). The life course approach considers these temporal aspects in analyzing whether and how different cohorts—defined as demographic groups influenced by shared opportunities, structures, and experiences—navigate life stages. In relation to educational trajectories, it is thus particularly relevant to study educational expansion involving societal and institutional change processes that shape the experiences of specific cohorts, in addition to considering individual educational decisions and attainment.

### 2.2 The educational expansion

Processes of educational expansion trace back to early educational institutions in ancient times, with significant development during the Enlightenment, early industrialization in the eighteenth century, and the establishment of national education systems with compulsory education by the late nineteenth century. The twentieth century, however, saw the most significant increase in educational expansion (Hadjar and Becker, 2009; Müller et al., 1997). The Cold War and the competition between capitalist and state-socialist countries played a significant role in the political demand for education. The Soviet Union's launch of the space orbiter Sputnik in 1957 was perceived as a major threat in the US and the Western world, leading to increased educational funding (Labaree, 2017). In Europe, concerns about falling behind in economic and technical progress led to demands for better exploitation of educational resources (Picht, 1964). Beyond economic arguments, the 1960s saw a push for education related to equality and participation in society. In Europe, Dahrendorf (1965) called for “education as a civil right,” viewing it as essential for dealing with political issues and societal participation (Dahrendorf, 1968). This socio-political perspective demanded educational reforms to abolish inequalities and increase participation (Hadjar and Becker, 2009). Hannum and Buchmann (2005) noted that global organizations like the UNESCO view educational opportunity expansion as a “win-win” strategy for promoting income equality and growth, reducing poverty, and laying the foundation for sustained economic growth and effective institutions (UNESCO, 2015).

This historical background, shaped by political, economic, and social debates, has significantly impacted the structure and accessibility of education. Evidence regarding educational expansion (Hannum and Buchmann, 2005; Hadjar and Becker, 2009) suggests that educational reforms have generally stimulated an increased demand for education and an increased number of graduates of upper secondary and tertiary education. Education as human capital is a central resource for individual and national economic development. Human capital theory (Becker, 1964) suggests that education is seen as a future investment pursuant to which higher skills and productivity rewarded by greater income. As long as economic growth and technological progress guarantee higher salaries, people aspire to higher educational degrees. The labor queue model (Thurow, 1975) recognizes that an applicant's education level signals their capacities, positioning them better in the labor market. As the average level of education increases, positional competition leads to individuals striving for even more education to distinguish themselves from others (Hirsch, 1976). Conflict and status group theories (Collins, 1971; Bourdieu, 1984; Bourdieu and Passeron, 1977) suggest that educational expansion is a result of conflicts between different status groups. More highly educated individuals are more likely to join dominant status groups, making educational qualifications increasingly important (Hadjar and Becker, 2009).

Although on the individual level education and income are strongly related, this universal finding does not apply at the macro level. Countries with a high percentage of more-highly educated workers but a discrepancy between supply and demand on the labor market cannot increase economic wealth by increasing education (Hannum and Buchmann, 2005). Benefits of educational expansion beyond monetary outcomes of education include political mobilization and rising political and reflection skills, value change, a longer life expectancy and healthier lifestyles (Hadjar, 2019).

### 2.3 Theorizing drivers of the educational expansion

The causes of educational expansion are rooted in political policies and reforms (Windolf, 1997), while the dynamics and momentum behind educational expansion differ by country.

In the Western world, educational expansion has been fuelled by increased skill demand, rising educational expenditures, and broader access to tertiary education (Becker and Blossfeld, 1991; Blossfeld and Hakim, 1997). Post-World War II educational policy measures in West Germany increased upper secondary general schooling, vocational schooling, and tertiary education institutions. Meanwhile, East Germany maintained a comprehensive schooling system until the collapse of the state-socialist German Democratic Republic (Hadjar and Berger, 2010).

A recent OECD (Organization for Economic Co-operation and Development) report “Trends Shaping Education 2025” (OECD, 2025) notes a broader set of drivers behind the ongoing educational expansion and the promotion of policies to increase societal educational levels. These drivers can be categorized into technological, economic, environmental, social, and political forces. Technological drivers include advances in artificial intelligence, virtual reality, and other technologies that transform teaching and learning and offer new opportunities for personalized learning and thus improved educational outcomes. Economic drivers relate to the classical argument mentioned above relating to the demand for a highly skilled workforce. As economies become more knowledge-based, there is a growing need for individuals with advanced skills and qualifications. Environmental drivers center around needs created by ecological crises and the need for sustainable development. The OECD report underscores the importance of educating students about environmental issues and equipping them with the knowledge and skills to address global challenges such as climate change. Social drivers contribute to inequality and polarization as social problems in societies, which are addressed by promoting inclusive education systems that cater to diverse populations and foster a sense of belonging and participation. Political drivers relate to geopolitical tensions and global conflicts that are perceived, by some, to be solvable by increased education to promote peace, stability, and international cooperation. An increase in educational level, from this perspective, fosters global citizenship and prepares students to navigate a complex and interconnected world. Moreover, Windolf's political theory of educational expansion highlights the role of political parties, parliaments, and state governments in negotiating education and deciding on expenditures and access (Windolf, 1997).

In summary, investment in education is widely regarded as a key response to various challenges and as an essential factor for societal progress. A well-educated society is considered necessary for innovation and economic growth, while also being seen as a means to reduce social inequality. The core assumption behind the latter is that greater access to education enables more people, regardless of social background, to attain higher qualifications, thereby promoting social mobility and reducing long term inequality.

Empirical research also supports investment in human capital through education as a crucial factor in economic development. However, the argument that expanding education alone can significantly reduce social inequality remains a subject of critical debate.

### 2.4 Conceptual approaches to educational inequalities

Educational inequalities refer to systemic variations in educational attainment structured by attributes such as social origin/class, gender, ethnicity, religion, or ability (Hadjar, 2019). Theorizing educational inequalities, primary, secondary, and tertiary effects may provide important conceptual tools. Boudon (1974) originally introduced his concept of primary and secondary effects in relation to inequalities in social background (e.g., social class, status). While primary effects relate to differences in educational achievement structured by social origin, as driven by differential resources (e.g., financial resources to pay additional lessons or educational material; social resources to receive support in learning), secondary effects relate to class-specific educational decisions based on cost-benefit evaluations against the background of benefits and costs of certain education alternatives (e.g., leaving school early vs. pursuing tertiary education). These decisions are influenced by perceived financial, social, and cultural resources. Tertiary effects have been recently added to this framework (e.g., Blossfeld et al., 2015; Esser, 2016). These refer to teacher evaluations of students and recommendations for students' educational pathways, often influenced by stereotypes about different social groups (e.g., working class vs. service class students, male vs. female students).

A similar argument can be derived from capital and social reproduction approaches (Bourdieu and Passeron, 1977; Bourdieu, 1986). These approaches examine class-specific social, economic, and cultural resources as well as socialized class-specific patterns of attitudes and behaviors toward education and schooling that advantage academic classes (or service classes) and disadvantage working class students in the uptake of education.

While educational expansion has led to a decrease and even reversal of gender inequalities in education (e.g., Becker, 2014), educational inequalities related to social background appear to be rather persistent (Blossfeld and Shavit, 1993; Breen et al., 2010; Hadjar and Uusitalo, 2016). This is because social inequalities are subject to lasting inter-generational secondary effects, namely class-specific perceptions of costs, benefits, and probabilities of future generations successfully finalizing a certain educational pathway (Becker, 2003). Two influential theses dominate empirical inquiries: The maximally maintained inequality (MMI) hypothesis (Raftery and Hout, 1993); and the effectively maintained inequality (EMI) thesis. The MMI hypothesis postulates that working classes benefit from educational expansion only after the educational desires of the upper middle classes have been saturated (Lucas, 2001). The EMI thesis suggests that even if educational institutions open up to all classes, advantaged classes seek distinction in quality, such as attending elite schools at higher rates. Relating to cultural reproduction approaches (Bourdieu and Passeron, 1977), inequalities persist as privileged groups take advantage of new educational opportunities to maintain their relative position in society.

The structure of education systems seems to have a profound impact on these mechanisms, impacting the change of educational inequalities during educational expansion. Studies have shown mixed results regarding the reduction of educational inequalities; some countries have seen a strong decrease in class-specific educational inequalities, while others have seen lower rates of reduction, particularly in stratified countries with strongly segregated education systems (e.g., Germany, Switzerland, the UK, and Hungary; Breen et al., 2010; Hadjar and Gross, 2016). This may be due to the fact that these highly stratified education systems, with early selection into different school tracks, foster class-specific educational decisions and lower probabilities that children from lower social backgrounds will attend upper secondary schooling (Van de Werfhorst and Mijs, 2010; Hadjar and Gross, 2016).

## 3 Context: education systems and educational expansion in Switzerland

The Swiss education system is highly decentralized, with around 90% of educational policies developed at the cantonal and local levels (Hega, 2000). Despite this, there have been significant efforts to harmonize institutional settings in the German-Swiss cantons and increase standardization. Swiss education systems, particularly in the German-speaking cantons, have a strongly stratified tradition (Buchmann et al., 2016); schooling is compulsory from ages 4/5 to 15 and students are grouped into different aspiration levels upon completing comprehensive primary schooling around the age of 12. The cantonal education systems in German-speaking regions are characterized, more so than the French or Italian-speaking regions, by the high value placed on vocational training in upper-secondary education. Vocational training is organized in terms of a dual system, combining apprenticeships in companies with vocational education at vocational schools and providing students with both theoretical knowledge and hands-on skills. The heterogeneity of schools is higher in Switzerland than in other countries with stratified education systems, where secondary schools include different tracks bound toward either general academic or vocational upper-secondary education. Certain areas, such as the city of Bern, are dominated by integrative school structures, in which students of different aspiration levels learn together. Furthermore, final track decisions regarding upper secondary academic or vocational education are not made before Grade 8 (Morinaj et al., 2017; Hadjar et al., 2021). In societies preferring education systems with both vocational and general educational tracks, the links between education and the labor market are strong, resembling the qualification-oriented education system type (Müller and Shavit, 1998). In such systems, formal educational qualifications have a strong role in determining occupational outcomes, as labor-market relevant knowledge is acquired in educational institutions. This type of system emphasizes the importance of educational credentials in the labor market, where qualifications serve as key indicators of an individual's skills and competencies.

Educational expansion in Switzerland took a slower course than in other Western countries, with a lower expansion rate of the upper-secondary general and tertiary education system. The links between social class, parental education, and educational attainment appear to be persistent in general (Buchmann et al., 1993, 2007), while there are also signs of a decreasing impact of social origin on educational attainment, benefiting disadvantaged social classes (Becker and Zangger, 2013; Hadjar and Uusitalo, 2016). The reason why Switzerland is perceived as a slow-expansion country in education, but at the same time maintains high knowledge levels and economic prosperity, may be due to the widespread participation in vocational post-secondary education, which already provides tertiary-level knowledge (Müller et al., 1997; Hadjar and Uusitalo, 2016).

Looking at the developments in more detail, Swiss education systems developed even before the core phase of the educational expansion in Europe in the 1960s (Hadjar and Becker, 2009). The introduction of compulsory education in 1874 was an important step in this development. However, there was never an overall project of “educational expansion” in Switzerland, not even in the “progressive” decades of the 1960s and 1970s. There is also no evidence of nationally or even regionally coordinated political control of such processes (Criblez and Magnin, 2001). There are, however, indications of a growth process that began around the mid-1950s, reached its peak in the 1960s, slowed down during the economic crises of the 1970s and 1980s, but continues to this day.

Similar to other countries, the two main drivers of the educational expansion in Switzerland were economic and social factors. On the one hand, since the mid-1950s, the business community has been insisting on measures to tackle the shortage of young skilled workers, initially focusing on the technical field. This lack of human capital, which extended to all areas of employment due to economic developments of the late 1950s and early 1960s, could only be solved through quantitative expansion and systemic reform. At the same time, demands were increasing for the democratization of the education system—or, more specifically, for a more equitable distribution of opportunities to pursue tertiary education between different social groups (between groups of different social and ethnic backgrounds, between urban and rural populations, between women and men and between groups of different religious backgrounds). This objective, captured in the slogan “more equal opportunities” in the education system, could only be realized through quantitative expansion coupled with systemic reform (Criblez and Magnin, 2001).

Education policy responded to demographic processes, labor market demands and political discourses by extending compulsory schooling—in Switzerland, through the nationwide introduction of nine compulsory school years. This expanded the system at the upper (upper secondary and tertiary levels) and lower end (kindergarten) of compulsory schooling. These reforms also introduced system differentiation, such as new types of grammar school and the differentiation of vocational training, along with the development and expansion of tertiary education. This included an initial push for institutionalizing continuing education and efforts to integrate different types of schools at the lower secondary level, all aimed at expanding access to tertiary education. In this way, tertiary education (grammar schools and universities) were opened up to broader sections of the population in a process of democratization.

All of these system changes continued—albeit at a slower pace and with smaller student cohorts—after the decline in economic prosperity. In the 1960s and 1970s, education policy experienced a “heyday” at communal, cantonal, and federal levels, leading to lasting changes in the form of constitutional amendments, comprehensive legislative processes, and new school buildings. Public spending on education rose sharply. The scholarship system expanded at unprecedented rates with the aim of democratizing tertiary education (Criblez and Magnin, 2001).

In summary, the main features of educational expansion in Switzerland in the last decades of the twentieth century and the first decades of the twenty-first century include the consolidation of compulsory education for children aged 6–15, with recent moves toward compulsory pre-schooling from the ages 4 or 5. There has also been a general standardization of schooling under the HarmoS Intercantonal Agreement 2007, a decreasing but still significant degree of stratification within the education system, with different educational track levels offering varying levels of permeability toward tertiary education, a strong dual system of vocational training combining classroom instruction with apprenticeships, and an increase in post-secondary education below university level, providing tertiary-level knowledge (Becker and Zangger, 2013; Schüpbach, 2014).

As we analyze a very complex research issue, considering different birth cohorts, trajectory patterns and inequalities along three axes of inequality, we refrain from posing a large number of hypotheses, but will postulate certain general expectations. Considering the conceptual considerations and the context of Switzerland, we expect:

(a) a prolongation of the core education phase in Switzerland over cohort succession;
(b) educational trajectory patterns that appear to be rather stable and center around the tracked education system with clusters strongly relating to general and higher academic education and clusters strongly relating to vocational education; and
(c) educational inequalities related to social background, gender, and migration background, which are notable across all birth cohort groups and lead to socially privileged classes and people without migration background having a higher likelihood of successfully achieving secondary and tertiary general (academic) education. Gender inequalities are expected to change profoundly over cohort succession.

## 4 Methods

### 4.1 Sample

As a data base we used the Swiss Household Panel (SHP; Tillmann et al., 2022). Since 1999 the survey collects detailed information on various aspects of life from individuals living in Switzerland on an annual basis. For our analysis, we focused on questions relating to educational pathways as well as social positions. As such, we used an initial set of questions addressing education qualifications currently being pursued or completed, intention to participate in further education, as well as gender. We then drew on a supplementary set of questions included in the individual survey between 2015 and 2017, which captured the timing of past educational achievements. Thirdly, we made use of retrospective life history data from a subsample (SHP_I) collected in 2001–2002, as well as data from the first wave of the refreshment sample (SHP_III) collected in 2013. This data provided information on educational trajectories. Finally, we included variables relating to social positions of parents, including nationality, level of education, and social class based on the Erikson-Goldthorpe-Portocarero (EGP) class scheme (SHP_SO).

We restricted our sample to individuals aged 15–40 who provided responses about their educational biographies. Additionally, we excluded right-censored individuals who had not yet reached the age of 40 by 2023 during the survey period. This exclusion removed all individuals born after 1983, as they cannot provide complete information on educational trajectories until the age of 40. Furthermore, we excluded individuals born before 1939, as there are very few individuals from these birth cohorts in the SHP. The final sample consisted of 2,580 respondents.

### 4.2 Operationalizations

For our sequence analysis, we traced educational trajectories beginning at secondary level II. We distinguished between 9 distinct states of secondary and tertiary education, which captured the diverse pathways individuals follow during this period. These states include: (1) compulsory school; (2) elementary vocational training (firm-based and school-based); (3) apprenticeship (CFC, EFZ level); (4) full-time vocational school (2–3 years), (5) general secondary education (2–3 years of general training, or 1 year in a school of commerce, au pair, or residential language courses), (6) maturity/higher entrance qualification (including vocational maturity and Teacher's Training College), (7) tertiary vocational education (with master's certificate, federal certificate), (8) technical or vocational school (including vocational high schools such as ETS, HTL, etc.), and (9) tertiary education (encompassing universities and academic high schools such as EPF or ETH, universities of applied sciences like HES or FH, universities of teacher education such as HEP or PH, and other tertiary specialized institutions). For a subsample, we also included information on *further education* as the 10th state.^1^

To investigate how social origin is associated with educational trajectories, we ran a multinomial logistic regression model. For this analysis, we used information about gender, migration background (measured as parents' nationality), social class, and parents' education. Gender (1—male, 0—female) and migration background (1—Swiss parent, 0—non-Swiss parents)^2^ are included as dummy variables. Social class is classified according to the EGP scale, distinguishing between: (I) upper service class; (II) lower service class; (III) routine non-manual employees; (IV) petite bourgeoisie (self-employed workers); and (V) the working class (skilled and unskilled workers), which also served as the reference category. Level of parents' education was divided into five levels: (1) compulsory education; (2) vocational education and training (elementary vocational training, apprenticeships, and full-time vocational school); (3) general secondary education (including maturity and higher entrance qualifications); (4) higher vocational education (tertiary vocational education and technical or vocational schools); and (5) tertiary education (universities and higher specialized institutions), with compulsory education as the reference category. For both social class and parental education, we consider the highest level attained by either the mother or the father.

Our analysis centers on the comparison of results for three different birth cohort groups, each representing a certain stage of educational expansion:

(a) 1939–1953: Individuals who completed primary education prior to educational expansion and started secondary level II (upper secondary education) either shortly before or at the very beginning of the expansion;
(b) 1954–1968: Individuals who began primary education at the start of the educational expansion period and transitioned to secondary level II during this period; and
(c) 1969–1983: Individuals who began secondary level II or tertiary education after the educational expansion period had already advanced.

To contextualize the comparisons made in subsequent analyses, a descriptive analysis of the three cohorts was conducted (see Table 1).

**Table 1:** Descriptive overview of cohort composition by gender, male, social class, and parent's education.

	**Cohorts 1939–1953 (%)**	**Cohorts 1954–1968 (%)**	**Cohorts 1969–1983 (%)**
**Gender**			
Male	46.27	44.51	44.38
Female	53.73	55.49	55.62
	100.00	100.00	100.00
**Migration background**			
No	95.01	95.86	95.94
Yes	4.99	4.14	4.06
	100.00	100.00	100.00
**Parents education**			
Compulsory school	19.61	15.93	10.43
Vocational education and training	48.74	52.3	53.68
General secondary education	9.97	10.59	8.59
Higher vocational education (tertiary VET, tech./voc. schools)	13.47	12.4	11.66
Tertiary education	8.21	8.78	15.64
	100.00	100.00	100.00
**Erikson-Goldthorpe-Portocarero (EGP) class scheme**			
V Working class (skilled and unskilled)	38.90	38.10	37.58
IV Petite bourgeoisie (self-employed)	27.96	22.14	12.74
III Routine non-manual employees	8.57	12.87	19.75
II Lower service class	12.06	12.28	15.92
I Upper service class	12.51	14.61	14.01
	100.00	100.00	100.00

### 4.3 Analytical strategy

As a statistical method, we applied sequence analyses to visualize and describe educational trajectories using annual data. The aim was to identify typical patterns of sequences in educational trajectories. This included considering various types of educational sequences at the secondary and tertiary levels, as well as their order and duration. To compare the (dis)similarities between sequences, we applied an optimal matching (OM) algorithm that quantifies the (dis)similarity of two sequences (Abbott, 1995). More specifically, we used a measure of how many transformations are required to change one sequence into another (MacIndoe and Abbott, 2004). As transformations we included substitution as well as insertion, and deletion.^3^ The resulting distance matrix, which represents the (dis)similarity between two sequences, is based on the minimum cost needed to transform one sequence into the other. For the cost calculation, standard optimal matching costs were used, with substitution costs set to 1 and insertion/deletion costs set to 0.5. After the optimal matching, the resulting dissimilarity matrix was used as the basis for hierarchical cluster analysis to group the sequences. Sequence and cluster analyses were conducted in R, using the TraMineR package (Gabadinho et al., 2011).

As a next step, Ward's hierarchical cluster analysis was applied to group the sequences. The goal was to identify typical patterns of educational trajectories by finding clusters that are as internally homogeneous as possible while maintaining significant differences between groups. It was important to avoid oversimplification caused by highly heterogeneous clusters, while too many small clusters may obscure typical patterns and compromise the goal of reducing complexity. To find the number of clusters we considered, on the one hand, the Average Silhouette Width index (ASW).^4^ On the other hand, the cluster solutions are evaluated qualitatively for interpretability and theoretical plausibility (Aisenbrey and Fasang, 2010, p. 433).

After identifying typical educational trajectories based on sequence analysis, we examined which socio-economic background variables were central to the assignment of individuals to specific educational trajectories. Next, we investigated the association between social origin as well as gender and educational trajectory clusters, using a nominal logistic regression, with the sequence clusters as the dependent variable (Cluster 1 serving as the reference category). We first ran nominal regression models including as the following factors as independent variables: gender; parents' nationality; and social class for each birth cohort group. Subsequently, we ran the same models, replacing social class with parents' education.

## 5 Results

### 5.1 Sequence analyses of secondary and tertiary educational trajectories

In this section, we examine the various educational trajectories and identify the typical educational paths that emerge empirically. Considering the changes in educational trajectories over time due to educational expansion, we also address these cohort differences by conducting the sequence analysis separately for the three birth cohort groups outlined above.

**Figure 1:**
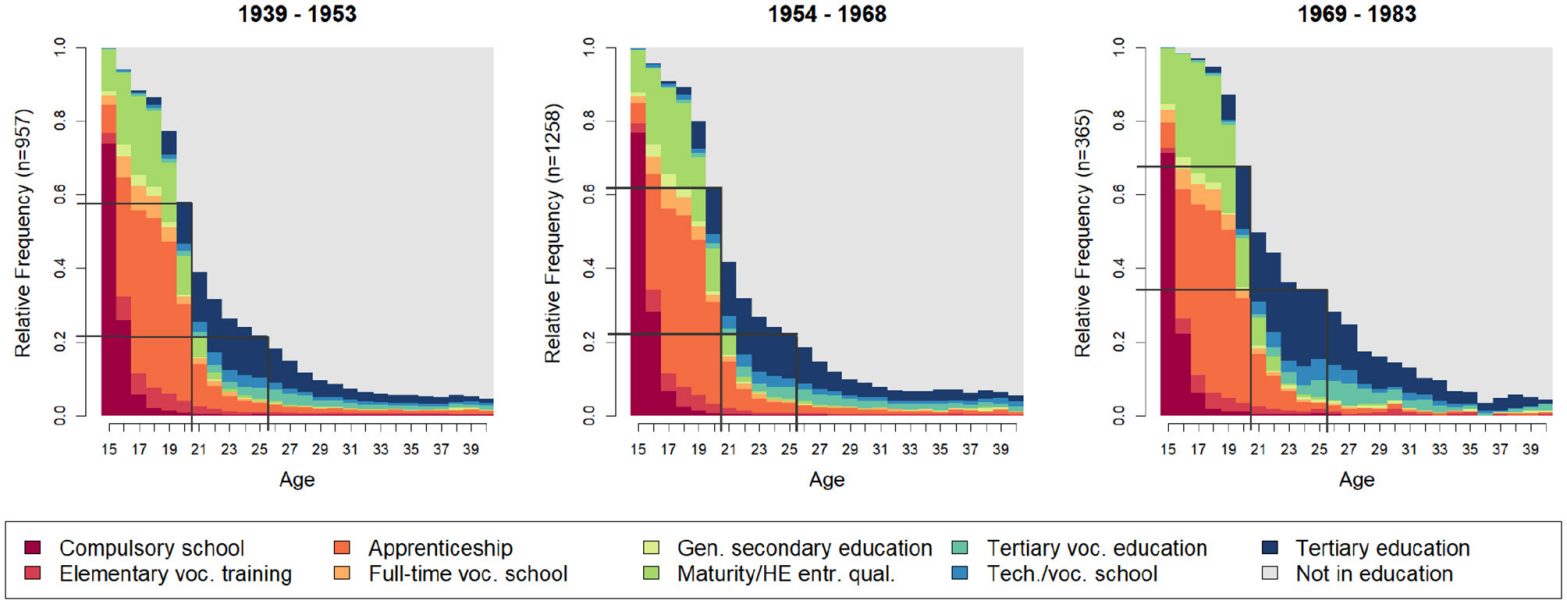
Sequence distribution plot of educational trajectories across birth cohort groups. Source: Swiss Household Panel data, 1999–2023.

In Figure 1, we first illustrate the distribution of educational attainment across three cohort groups, beginning at secondary and tertiary education. Notably, the extension of the education and training phase is particularly evident between the cohorts born between 1954 and 1968, who began their secondary and tertiary education during the period of educational expansion, as well as those born between 1969 and 1983, who started their education after the expansion had already progressed.

Visually, this is most clearly reflected in the expansion of the dark blue bars, representing tertiary education. Additionally, the lighter blue bars, which represent tertiary vocational education and technical or vocational schools, also show an increase (see also Figure A.2). Along with this growth, the overall length of the education phase has increased. The cohorts born between 1939 and 1953, as well as those born between 1954 and 1968, show that around 80% had completed their education prior to the age of 25. However, for the 1969–1983 cohort, only 65% had completed their education before that age.

Differences between the cohort groups also appear in the early 20s. Both the 1939–1953 and 1954–1968 cohorts show that around 40% had completed their education prior the age of 20. However, for those born between 1969 and 1983, only 32% had completed their education before that age.

Having developed a general understanding of the distribution and duration of sequences within educational trajectories, we now proceed with the sequence analysis. The primary objective is to identify typical clusters of educational trajectories and, subsequently, to evaluate their significance across the three birth cohort groups, as well as to reveal changes in sequence patterns between these groups. Based on ASW and most of the other cluster performance indices, the two-cluster solution appears optimal across the three cohort groups (Figure A.3).

Figure A.4 refers to the two-cluster solution. It illustrates the educational trajectory clusters for the different birth cohort groups. The relative frequencies are displayed on the y-axis, with the number of cases belonging to each cluster indicated in brackets, and the age of individuals at the different stages of educational trajectories is shown on the x-axis.

Figure A.4 clearly shows that the two-cluster solution mainly separates individuals into two distinct groups: individuals who follow a more vocationally oriented educational path and those who pursue tertiary education after obtaining their Matura (high school diploma). Considering the distribution of individuals within the two clusters, we find that most individuals across all cohort groups belong to Cluster 1, representing those who pursue a vocationally oriented education. This accounts for approximately 80% of the 1939–1953 and 1954–1968 birth cohorts, while for the 1969–1983 cohorts this share decreases to around 70%.

**Figure 2:**
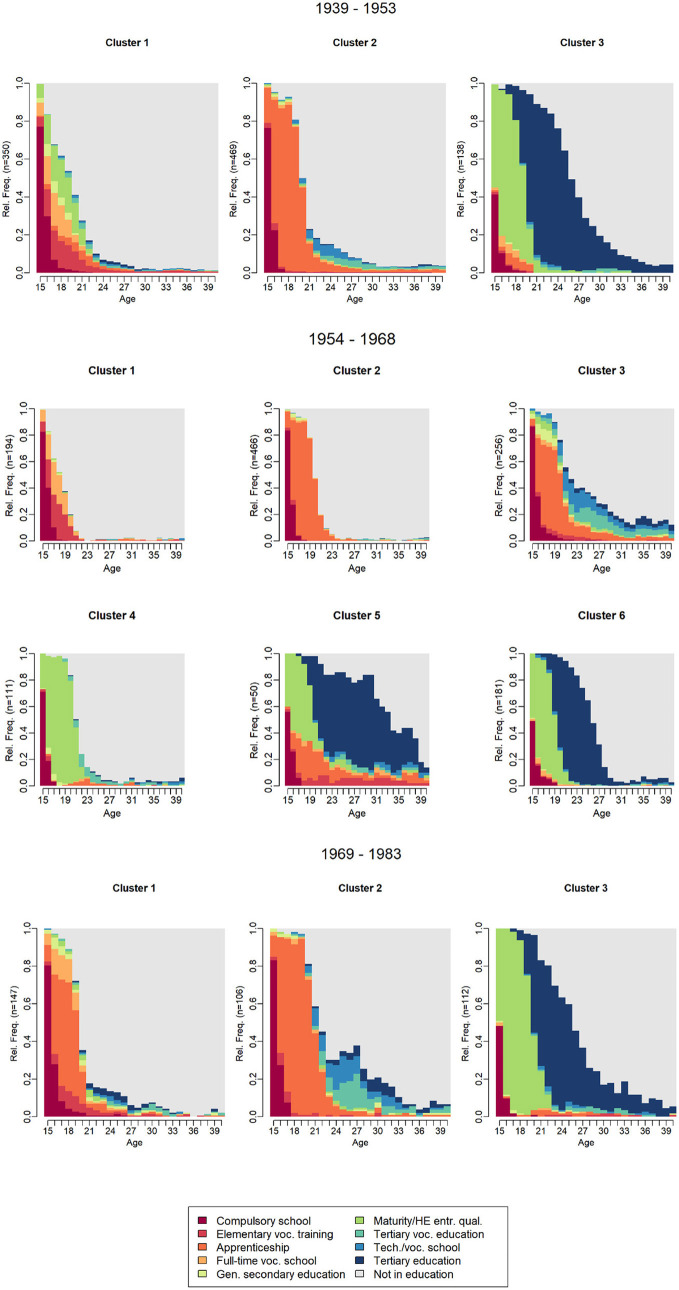
Educational trajectory clusters—state distribution plots across birth cohort groups. Source: Swiss Household Panel data, 1999–2023.

However, due to high heterogeneity, particularly within the first cluster, we ultimately choose—according to the ASW index—the second-best solution (Figure A.3): a three-cluster solution for the 1939–1953 cohorts, a six-cluster solution for the 1954–1968 cohorts, and a three-cluster solution for the 1969–1983 cohorts, as presented in Figure 2.

In Figure 2, shifts in educational pathways over time are reflected in the changes in cluster formation across the three birth cohort groups.

Over time, the group continuing with elementary vocational training or vocational school after compulsory education has lost its distinct cluster status. While in the 1939–1953 cohort, Cluster 1, and in the 1954–1968 cohorts, Clusters 1 and 4 represent individuals who follow this path—some even obtaining a (vocational) Matura before entering the labor market—this group no longer forms a distinct cluster in the 1969–1983 cohorts.

The structure of apprenticeship-based career paths has become more differentiated across cohorts. Cluster 2 in the 1939–1953 cohorts is associated with apprenticeships. In later cohorts (1954–1968 and 1969–1983), this cluster splits into two groups: one (Cluster 2 for the 1954–1968 cohorts and Cluster 1 for the 1969–1983 cohorts) undergoes a shorter apprenticeship and typically enters the labor market directly afterward, while the other (Cluster 3 for the 1954–1968 cohorts and Cluster 2 for the 1969–1983 cohorts) pursues a longer apprenticeship, often followed by tertiary vocational education, a master's degree, or further studies at a technical or vocational school, representing a significant increase in years of education.

The pathway to tertiary education has expanded over time, with more individuals pursuing university degrees after obtaining their Matura. The final clusters across all cohorts (Cluster 3 for 1939–1953 and 1969–1983, as well as Clusters 5 and 6 for 1954–1968) represent individuals who took this route, with many completing their education in their mid-to-late 20s. In the 1939–1953 cohorts, a small subgroup within Cluster 3 first completed an apprenticeship before obtaining their Matura and continuing to tertiary education. This subgroup became a distinct cluster (Cluster 5) in the 1954–1968 cohorts but disappeared in the 1969–1983 cohorts.

In sum, the largest group still consists of individuals who complete an apprenticeship and enter the labor market around the age of 21, either directly or after one to two additional years of schooling. However, there has been a noticeable increase in those pursuing tertiary vocational education—both in terms of the frequency and duration of participation in advanced vocational programs and enrolment in technical/vocational schools following an apprenticeship. In contrast, the group continuing with basic vocational training or vocational school after compulsory education has declined in significance.

Additionally, the number of individuals obtaining a Matura followed by tertiary education has increased: in the 1939–1953 cohorts, Cluster 3 accounted for 14.4%; in the 1954–1968 cohorts, Clusters 5 and 6 made up 18.4%; and in the 1969–1983 cohorts, Cluster 3 represented 30.7%.

### 5.2 Sequence analysis including further education

When including further education training courses in the sequence analysis for the cohorts from 1974 to 1983, the three-cluster solution proves to be both supported by the ASW values and meaningful. Similar to the previous three-cluster solution for the 1969–1983 birth cohorts, comparable patterns of sequence trajectories emerge. The first two clusters consist of individuals following a vocationally oriented trajectory, differentiated by the length of their apprenticeships—Cluster 1 includes those with shorter apprenticeships, while Cluster 2 comprises those with longer apprenticeships. The third cluster consists of individuals who have completed a Matura (university entrance qualification) followed by tertiary education.

**Figure 3:**
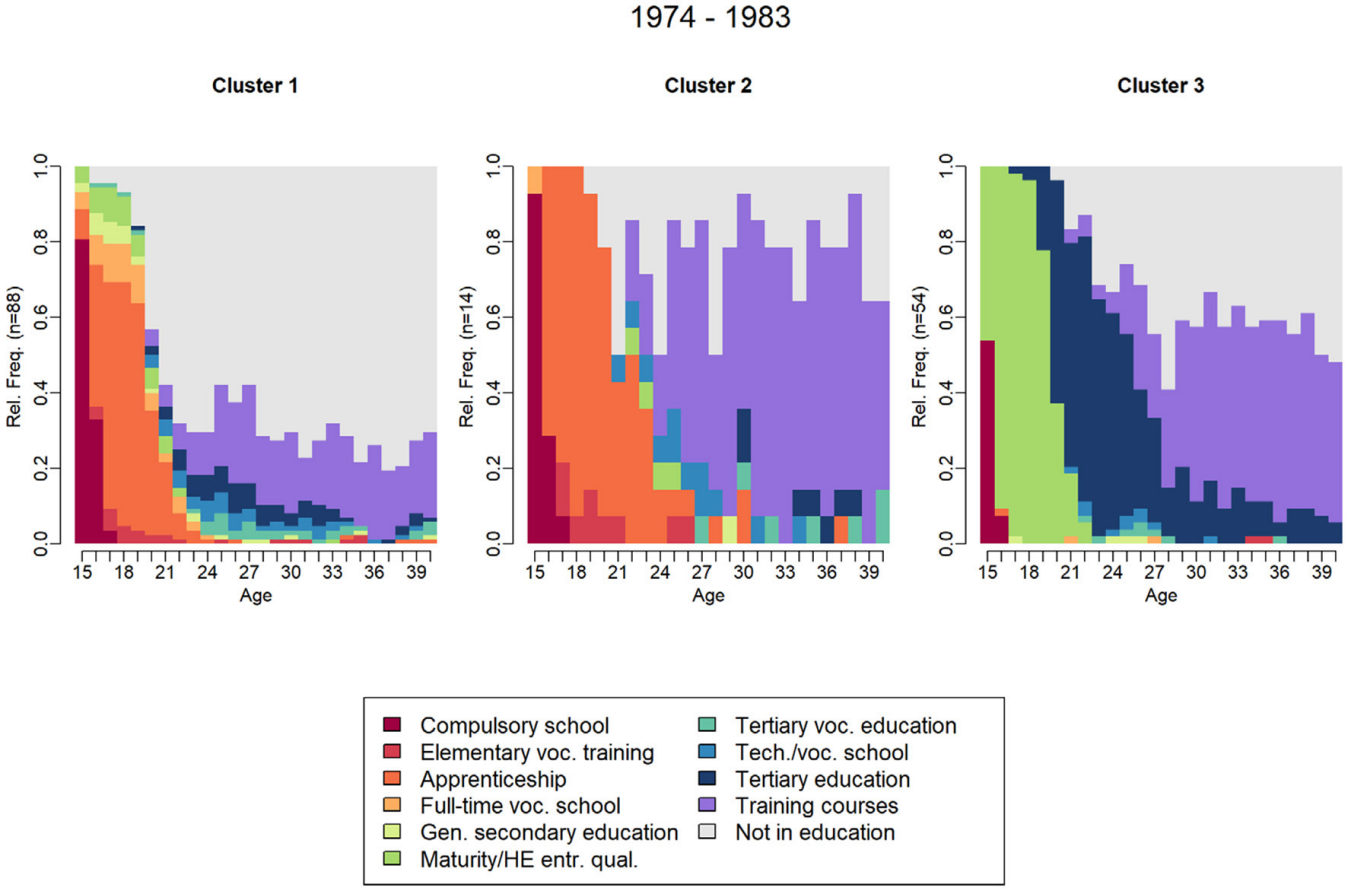
Educational trajectory clusters n- state distribution plots across birth cohort groups including further education. Source: Swiss Household Panel data, 1999–2023.

In relation to further education, Figure 3 shows that individuals in Cluster 3 (34.62%), who have completed tertiary education, engage in further education to a significant extent and often repeatedly. In contrast, this is observed in only a small subset of individuals with a vocationally oriented education (Cluster 2, 8.97%), while the majority of individuals with a vocational education background (Cluster 1, 56.41%) participate in further education only occasionally, typically every 5–6 years.

### 5.3 The role of social origin, gender, and migration background in shaping educational trajectories

**Figure 4:**
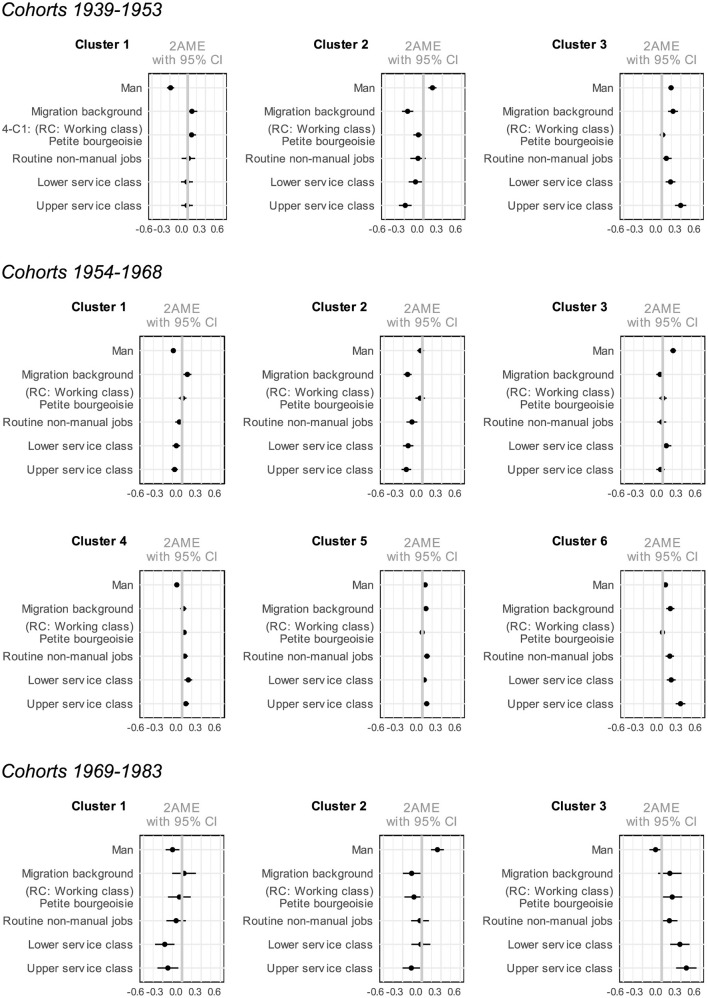
Average marginal effects of multinomial logistic regression on clusters by gender, migration background, and social class.

**Figure 5:**
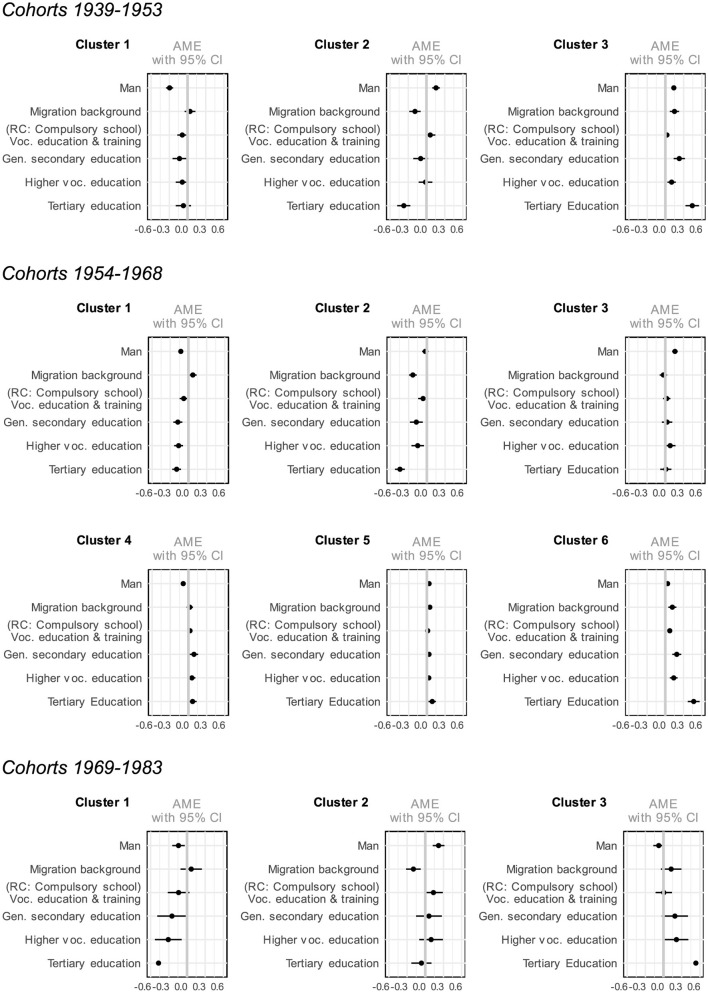
Average marginal effects of multinomial logistic regression on clusters by gender, migration background, and parents' education.

In Tables A.2–A.7, multinomial logistic regression models show the relationship between social economic background and educational trajectories across the three different birth cohort groups. Results are depicted in Figure 4 (gender, migration background, social class) and Figure 5 (gender, migration background, parents' education).

Parental education, social class, gender, and migration background are all associated with membership in specific educational trajectory clusters. Moreover, the regression model results remain largely consistent across the latter two variables. This means that whether social origin is measured as social class alongside gender and parental nationality or replaced by parental educational attainment, the overall patterns and relationships observed in the model remain stable. This finding suggests that both social class and parental education play a comparable role in shaping educational trajectories.

Looking at the variables measuring the effects of social origin, individuals from higher social classes and those with more highly educated parents—especially those from the upper service class with tertiary-educated parents—are less likely to be in clusters associated with apprenticeships without Matura or elementary vocational training (such as Cluster 2 for the 1939–1953 and 1954–1968 birth cohorts, and Cluster 1 for the 1954–1968 and 1969–1983 birth cohorts). These individuals are characterized by longer educational trajectories leading to tertiary education, and are more likely to come from higher social classes and have parents with higher levels of education (Cluster 3 for the 1939–1953 and 1969–1983 birth cohorts, and Clusters 5 and 6 for the 1954–1968 birth cohorts).

Social background, however, appears to have no influence on participation in tertiary vocational education, as well as technical or vocational schooling after an apprenticeship (Cluster 3 for the birth cohorts 1954–1968 and Cluster 2 for the birth cohorts 1969–1983). This suggests that, on one hand, such trajectories offer opportunities for social mobility for individuals from lower social classes and those with parents who have lower levels of education. On the other hand, these trajectories provide individuals from higher social classes who did not follow a typical status-preserving educational path with an alternative route to maintaining their social position through continued education after an apprenticeship.

In relation to gender, the findings indicate that women are more likely to belong the cluster characterized by shorter educational pathways, typically involving elementary vocational training or vocational school after compulsory education (Cluster 1 in the 1939–1953 and 1954–1968 birth cohorts). In contrast, men are more likely to follow educational trajectories that include longer apprenticeships, often followed by tertiary vocational education or technical/vocational schools (Cluster 2 for the 1939–1953 and 1969–1983 birth cohorts, as well as Cluster 3 for the 1954–1968 birth cohorts). Amongst earlier cohorts, individuals are also more likely to pursue a Matura followed by tertiary education (with Cluster 3 being significant only for the 1939–1953 birth cohort). This trend demonstrates that, while the gender gap in clusters primarily involving elementary vocational training, vocational school, and apprenticeships remains stable across cohorts, the difference in trajectories leading to tertiary education has decreased over time. This aligns with recent research (Becker, 2014) and statistics (e.g., BFS 2023), which indicate that women in younger cohorts are increasingly pursuing higher educational pathways and are even slightly overrepresented in university institutions.

Migration background also plays a role in shaping educational trajectories. The results indicate that individuals whose parents do not hold Swiss citizenship are more likely to pursue educational paths leading to higher qualifications,^5^ although this trend becomes insignificant in the most recent cohorts. Furthermore, a distinction can be seen between Clusters 1 and 2; individuals who opt for longer apprenticeships (in Cluster 2 across all birth cohorts) are more likely to have no migration background^6^ than those who predominantly choose shorter vocational training or vocational school after compulsory education (Cluster 1 across all birth cohorts).

In summary, the results indicate a decrease in gender inequalities and a positive association between migration background and educational trajectories leading to higher educational outcomes. Additionally, belonging to clusters with higher levels of educational attainment is strongly associated with both higher parental education and higher social class across different birth cohorts. This highlights the importance of social origin in shaping educational pathways, with higher social class and parental education facilitating access to longer and more advanced educational trajectories.

## 6 Discussion and conclusions

The results support expectation (a) by demonstrating a prolongation of the education phase between the two cohorts of those born between 1954 and 1968 and those born between 1969 and 1983. This prolongation is particularly evident in relation to upper-secondary vocational and tertiary education. Initially, a two-cluster solution identified a typical vocationally oriented trajectory (centered on upper secondary vocational education) and a typical academically oriented trajectory (centered on general upper-secondary education and tertiary education). This clearly resembles stratified and externally-differentiated education systems with dual pathways and low permeability between different tracks (Hadjar and Gross, 2016; Van de Werfhorst and Mijs, 2010), thus meeting expectation (b). To account for heterogeneity, we chose a three-cluster solution for 1939–1953, a six-cluster solution for 1954–1968, and a three-cluster solution for 1969–1983. These clusters, however, are again shaped by the stratified education systems: one pattern centered around upper-secondary general and tertiary education, while another pattern centered around vocational upper-secondary education and comparably early entry in the labor market. Clusters beyond the duality relate to trajectories that include both vocational upper-secondary education and vocational or even general tertiary education. Over time, the group continuing with basic vocational training lost distinct cluster status, as apprenticeship-based career paths became more differentiated. The pathway to tertiary education expanded, with more individuals pursuing university degrees after obtaining their Matura. Bringing in further education in terms of lifelong learning, a three-cluster solution for the 1974–1983 cohorts showed similar patterns. A third cluster developed around tertiary education graduates who engaged significantly in further education, while vocational education clusters emerged less frequently. All in all, the study reveals shifts in educational trajectories, with an increase in tertiary vocational education and university degrees over time.

As the study does not include an international comparison, we cannot judge whether the educational expansion took a slower course in Switzerland than in other countries. However, it becomes clear that a certain educational expansion has taken place and that—if analysis allows for heterogeneity—educational trajectories beyond the typical duality in stratified education systems have emerged.

Expectation (c) related to social origin, gender, and migration background as factors impacting typical educational trajectory patterns. The results indicate, on the one hand, a decline in gender inequality in educational trajectories that lead to a tertiary degree. Migration background is positively associated with tertiary education and negatively associated with vocational-oriented educational trajectories, with weakening effects over time. Moreover, the results show that social inequality exists in educational trajectories; while a typical vocationally-oriented educational trajectory involving apprenticeship is more commonly chosen by individuals from lower social classes with parents who have lower educational backgrounds, tertiary education tends to be chosen by individuals from higher social classes with parents who have higher educational qualifications. However, individuals who go on to pursue a tertiary vocational degree after completing an apprenticeship do not show any effects related to social origin. This finding indicates that individuals from lower social backgrounds can compensate for earlier educational deficits in their mid-20s by continuing their education through tertiary vocational training or technical/vocational schooling, thereby mitigating the educational inequality resulting from stratified education systems with early selection. This suggests that these educational pathways do not provide a means of upward social mobility for those from disadvantaged backgrounds. The findings also highlight a route for status preservation for those from more privileged backgrounds, as individuals from higher social backgrounds who begin their educational trajectory in a way that does not align with their social status can use this pathway to attain higher education later in their educational trajectory.

Some limitations of the study include a limited period time frame, examining educational trajectories only until the age of 40. Ideally, these analyses would cover work trajectories up to retirement age. However, this would introduce a right-censoring problem due to missing data resulting from panel attrition. Additionally, since the Swiss Household Panel only includes people from the age of 15, earlier educational stages could not be included. As such, our study was unable to account for the prolongation of compulsory education through early childhood education and pre-schooling. Future studies may compare different countries' educational trajectories, noting that internally comparable panel data remains valuable.

In addition to sequence analysis for specific cohort groups, future research could benefit from an age-period-cohort (APC) analysis. Sequence analysis is effective at visually representing multiple states simultaneously to uncover life course patterns. However, the APC approach could provide a more detailed examination by focusing on individual educational states and disentangling the effects of age, historical period, and cohort through regression-based modeling techniques. In conclusion, in the Swiss context, educational expansion encompasses not only the growth of tertiary education but also the significant development of vocational education at upper-secondary and tertiary level. Additionally, this study demonstrates that social origin has a significant and persistent effect on educational trajectories across cohorts, indicating that educational disadvantages are often inherited. However, the possibility of achieving a higher educational qualification through a vocationally oriented educational trajectory suggests that early disadvantages can be overcome, thereby reducing educational inequality in stratified systems.

## Data Availability

Publicly available datasets were analyzed in this study. This data can be found here: https://forscenter.ch/projects/swiss-household-panel.

## Footnotes

^1^Information on further education has only been collected through the annual Individual Core Questionnaire from 1999 to 2023. This means that for the 1974–1983 cohorts, this information can be included in the analyses starting at latest from the age of 25.

^2^Since too few people are classified as non-Swiss when measuring one's own nationality, resulting in a too-small cell population, we use the nationality of the parents instead. The category “Swiss parent” refers to having at least one Swiss parent, while “non-Swiss parents” means that neither of the two parents is Swiss.

^3^Since both the order and duration of sequences are crucial to our study, we use a distance measure that is sensitive to both aspects (Aisenbrey and Fasang, 2010).

^4^ASW evaluates the cohesion within a cluster and the separation between clusters, with higher values indicating better clustering.

^5^These differences are also consistent with the BFS (2023) statistics.

^6^Or at least one Swiss parent.
