# Supplementary material for: Educational trajectories within and beyond the core education phase in Switzerland: a sequence analysis based on SHP data 1999–2023
Source: Front Sociol. 2025 Jun 5;10:1585910. doi: 10.3389/fsoc.2025.1585910 (PMC12177887; doi:10.3389/fsoc.2025.1585910)
Supplement: Supplementary file 1 [file Data_Sheet_1.docx]

| **Appendix**  **Figure A.1: Sequence index plot of educational trajectories across cohort groups** |
| --- |
| 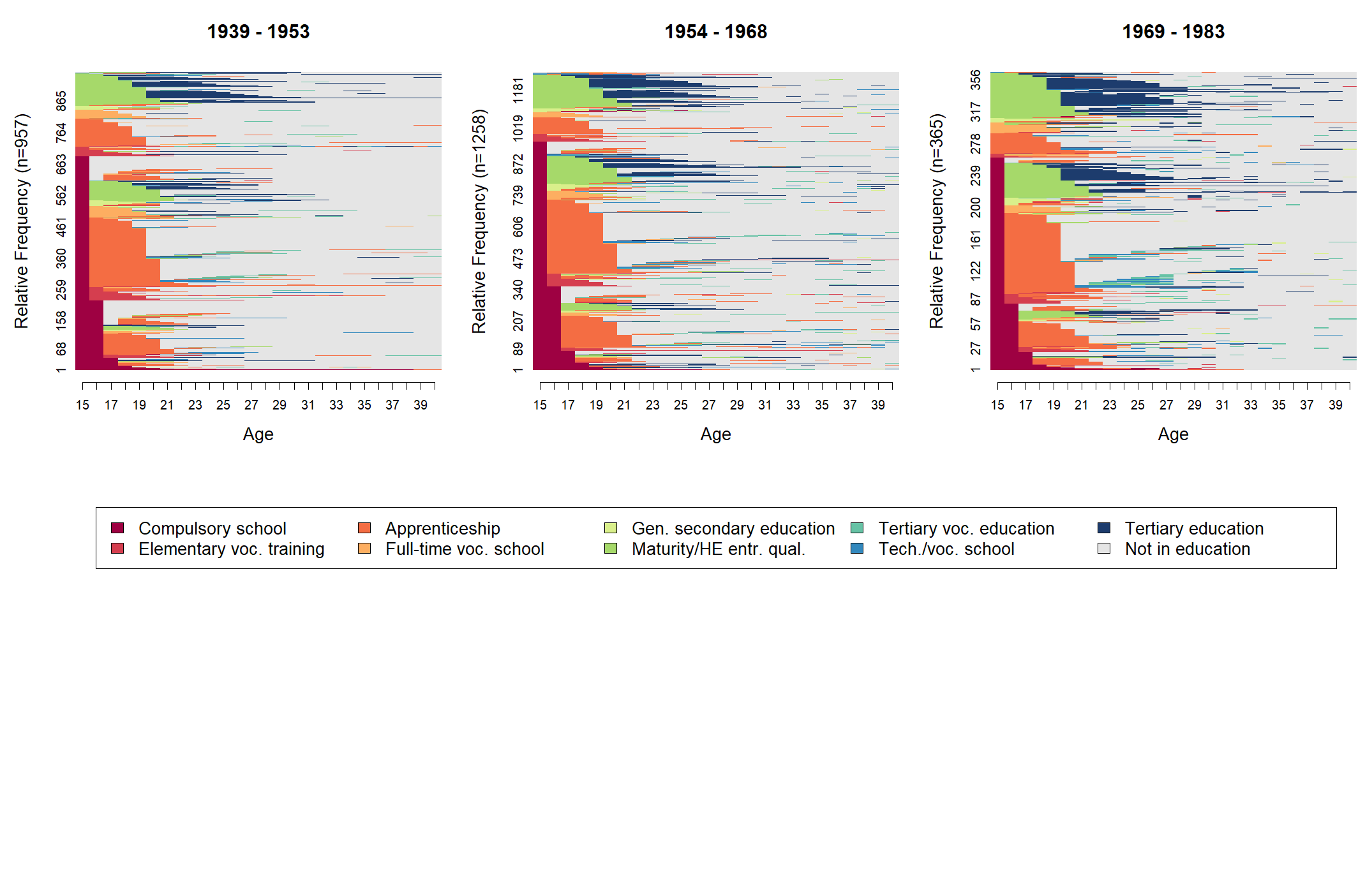 |
| *Source: Swiss Household Panel data, 1999–2023* |

| **Figure A.2: Mean time plot of the different educational states across cohorts** |
| --- |
| 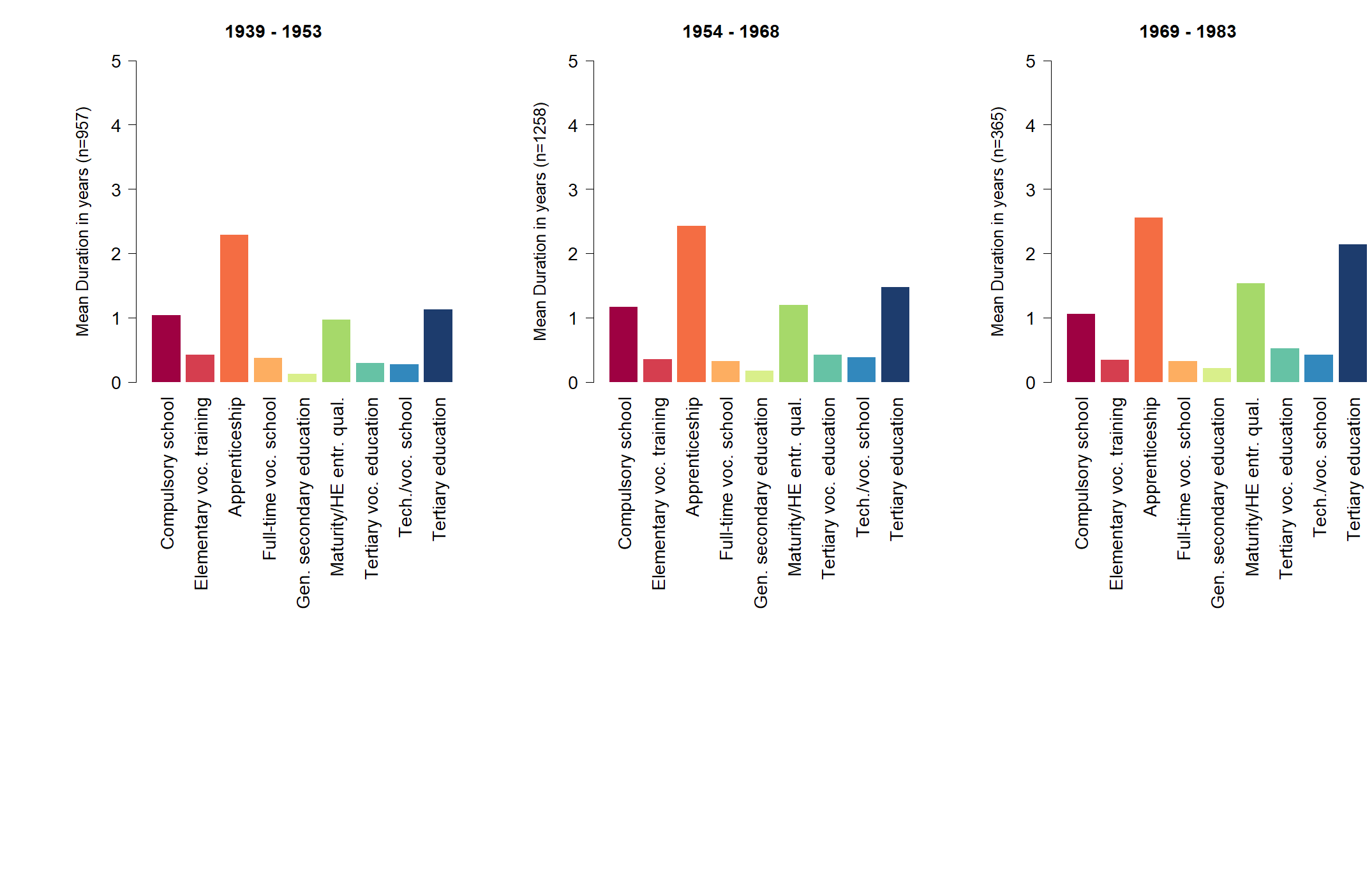  *Source: Swiss Household Panel data, 1999–2023* |

| **Figure A.3: Cluster performance plots** | | |
| --- | --- | --- |
| 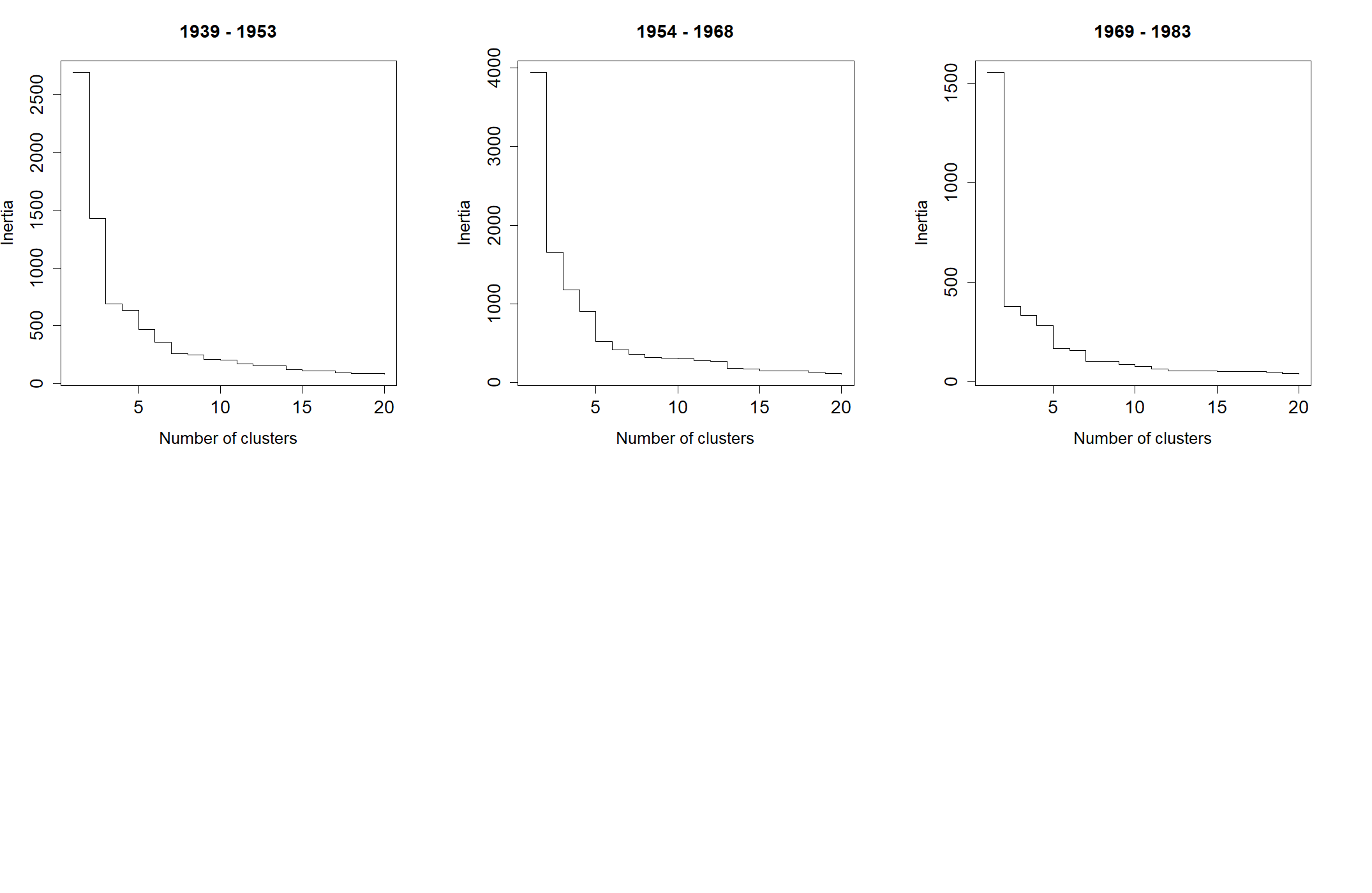 | | |
| 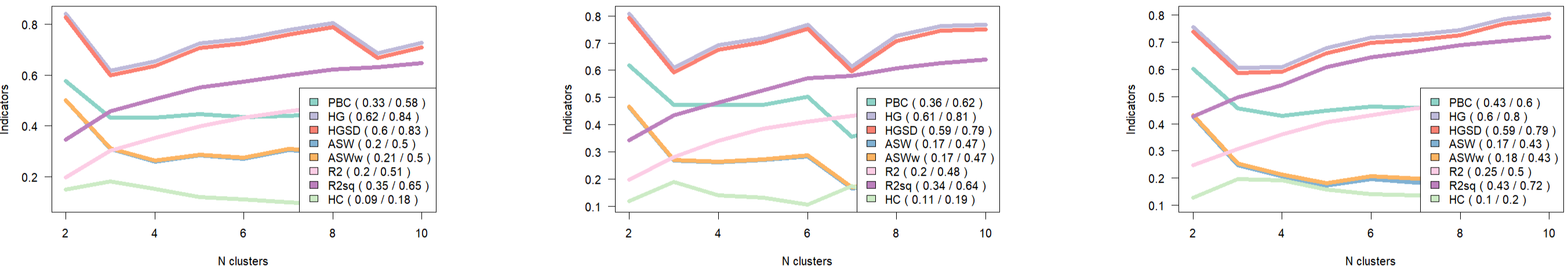 | 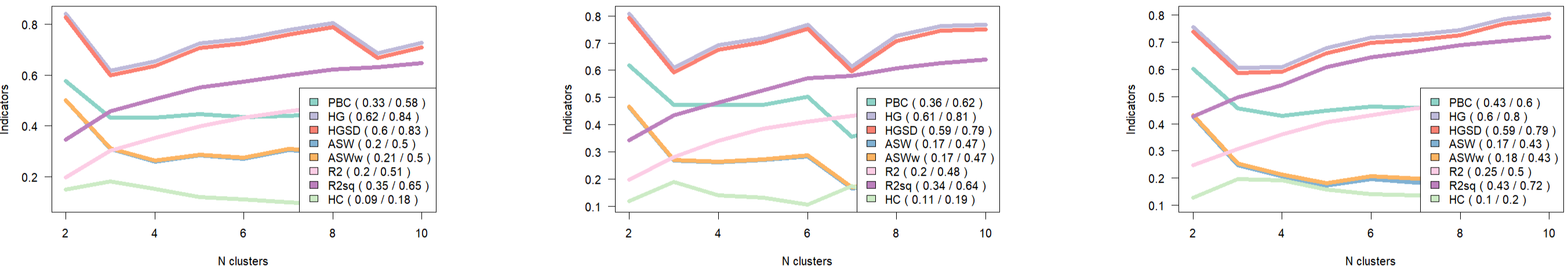 | 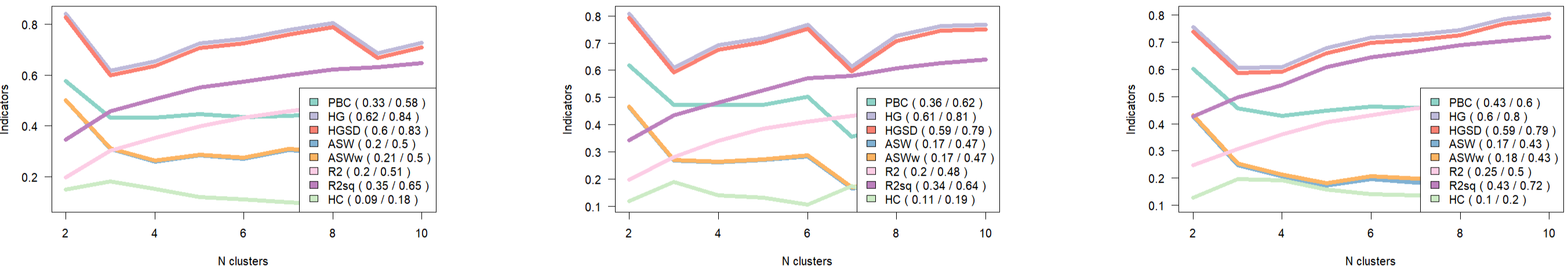 |

| **Figure A.4: Educational trajectory clusters - state distribution plots across cohort groups**  *2-Class Solution* |
| --- |
| 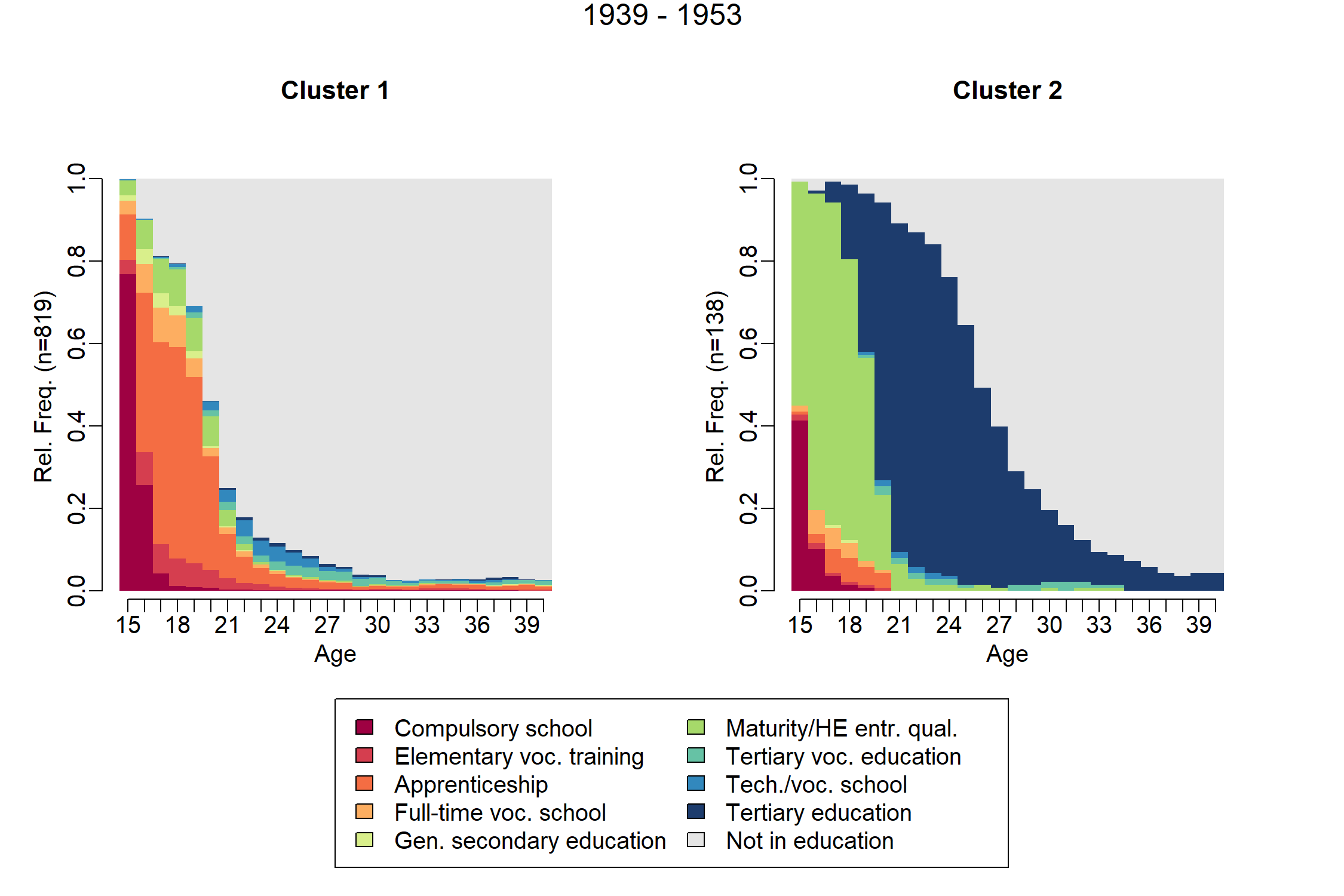 |
|  |
| 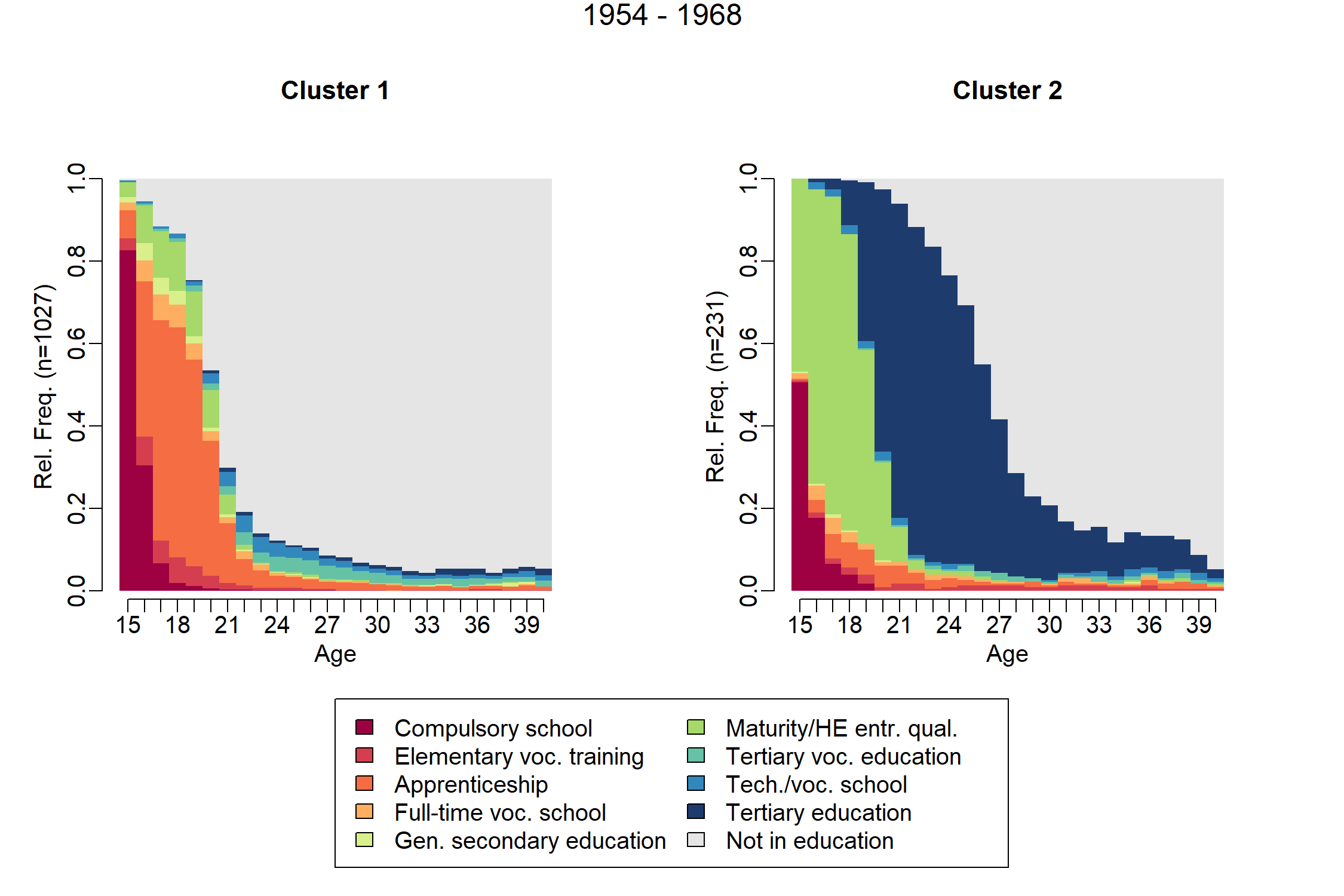 |
| 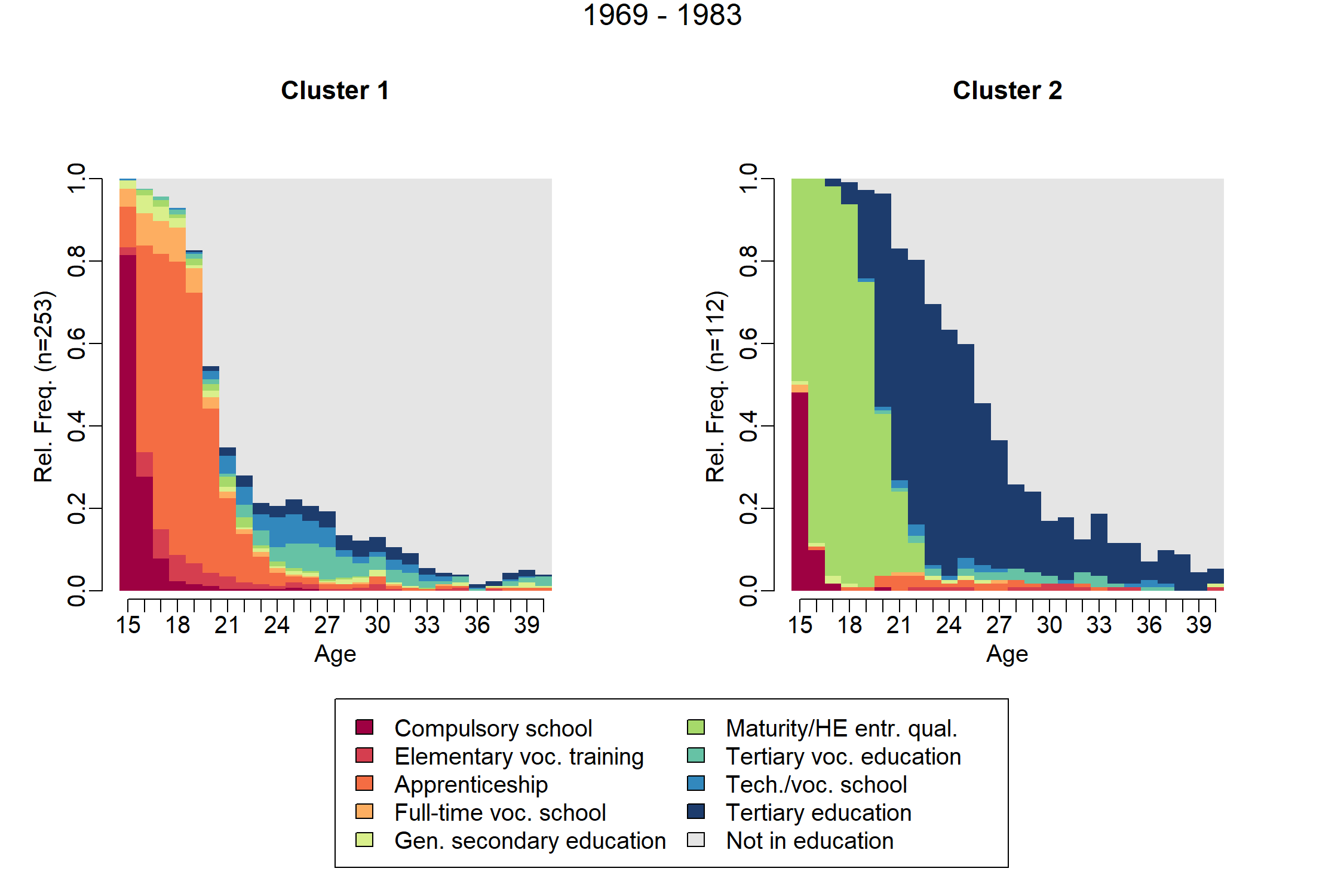 |
| *Source: Swiss Household Panel data, 1999–2023* |

| **Figure A.5: Educational trajectory clusters - state distribution plots across cohort groups**  *4 to 7 Class Solution* |
| --- |
| 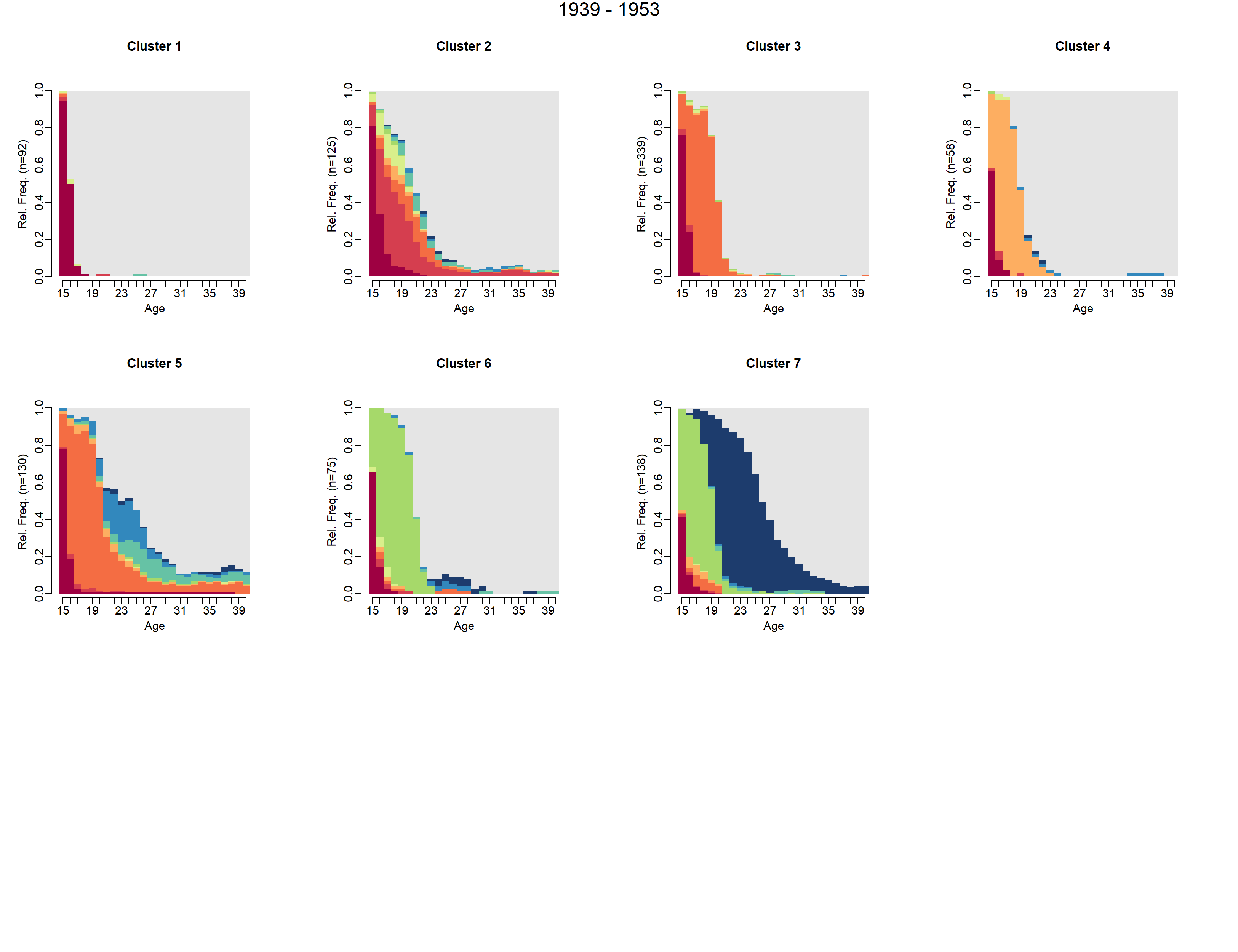 |
| 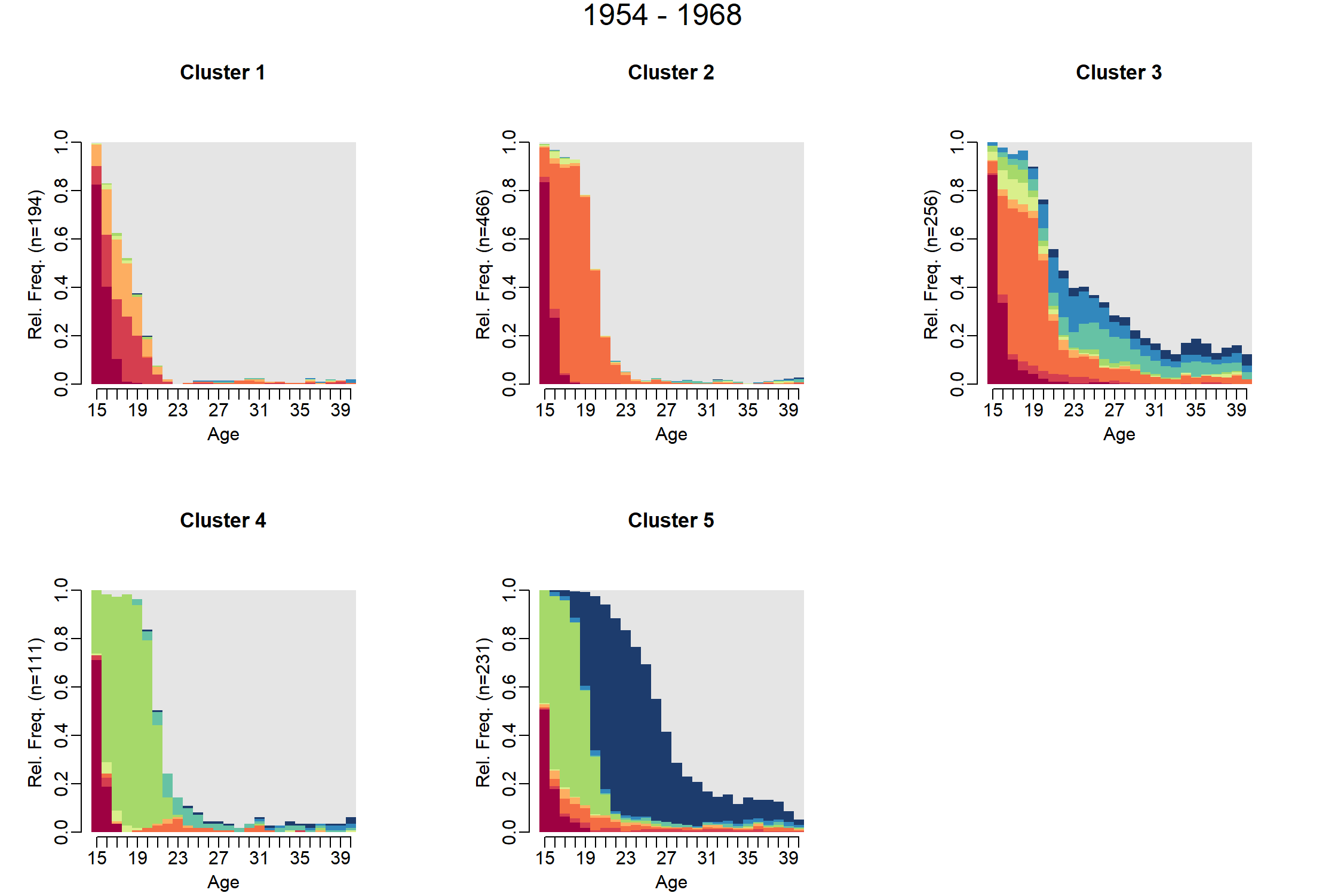 |
| 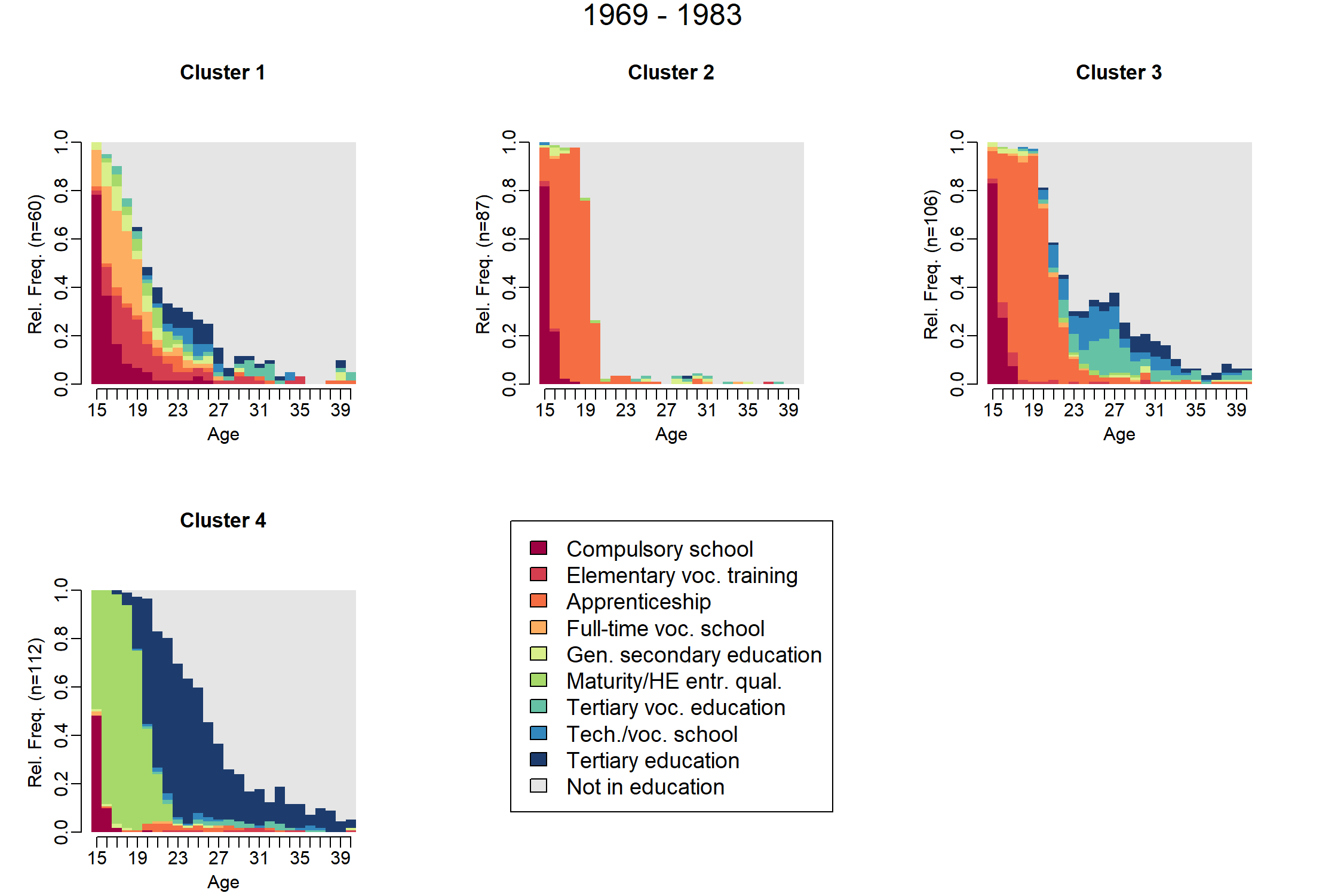  *Source: Swiss Household Panel data, 1999–2023* |

| \| **Table A.1: Quality indices for the four best solutions** \| \| \| \| \| \| \| \| \| \| \| --- \| --- \| --- \| --- \| --- \| --- \| --- \| --- \| --- \| --- \| \|  \|  \| N groups \| Index \| N groups \| Index \| N groups \| Index \| N groups \| Index \| \| 1939 - 1953 \| PBC \| 2 \| 0.577 \| 8 \| 0.448 \| 5 \| 0.446 \| 7 \| 0.440 \| \| HG \| 2 \| 0.842 \| 8 \| 0.805 \| 7 \| 0.777 \| 6 \| 0.743 \| \| HGSD \| 2 \| 0.829 \| 8 \| 0.788 \| 7 \| 0.760 \| 6 \| 0.726 \| \| ASW \| 2 \| 0.501 \| 3 \| 0.310 \| 7 \| 0.306 \| 8 \| 0.295 \| \| ASWw \| 2 \| 0.502 \| 3 \| 0.312 \| 7 \| 0.310 \| 8 \| 0.300 \| \| CH \| 2 \| 234.425 \| 3 \| 206.169 \| 4 \| 172.597 \| 5 \| 157.529 \| \| R2 \| 10 \| 0.511 \| 9 \| 0.496 \| 8 \| 0.477 \| 7 \| 0.459 \| \| CHsq \| 2 \| 505.698 \| 3 \| 402.452 \| 4 \| 325.042 \| 5 \| 292.795 \| \| R2sq \| 10 \| 0.648 \| 9 \| 0.630 \| 8 \| 0.623 \| 7 \| 0.599 \| \| HC \| 8 \| 0.089 \| 7 \| 0.099 \| 6 \| 0.111 \| 5 \| 0.119 \| \|  \|  \| \| \| \| \| \| \| \| \| \|  \|  \| N groups \| Index \| N groups \| Index \| N groups \| Index \| N groups \| Index \| \| 1954 - 1968 \| PBC \| 2 \| 0.618 \| 6 \| 0.503 \| 3 \| 0.473 \| 4 \| 0.472 \| \| HG \| 2 \| 0.808 \| 10 \| 0.768 \| 6 \| 0.768 \| 9 \| 0.764 \| \| HGSD \| 2 \| 0.793 \| 6 \| 0.752 \| 10 \| 0.750 \| 9 \| 0.745 \| \| ASW \| 2 \| 0.465 \| 6 \| 0.284 \| 5 \| 0.271 \| 3 \| 0.269 \| \| ASWw \| 2 \| 0.466 \| 6 \| 0.287 \| 5 \| 0.273 \| 3 \| 0.271 \| \| CH \| 2 \| 309.708 \| 3 \| 245.236 \| 4 \| 215.309 \| 5 \| 196.293 \| \| R2 \| 10 \| 0.482 \| 9 \| 0.466 \| 8 \| 0.450 \| 7 \| 0.432 \| \| CHsq \| 2 \| 652.838 \| 3 \| 482.087 \| 4 \| 387.285 \| 5 \| 348.754 \| \| R2sq \| 10 \| 0.639 \| 9 \| 0.627 \| 8 \| 0.606 \| 7 \| 0.580 \| \| HC \| 6 \| 0.106 \| 10 \| 0.113 \| 9 \| 0.116 \| 2 \| 0.120 \| \|  \|  \| \| \| \| \| \| \| \| \| \|  \|  \| N groups \| Index \| N groups \| Index \| N groups \| Index \| N groups \| Index \| \| 1969 - 1983 \| PBC \| 2 \| 0.601 \| 8 \| 0.464 \| 6 \| 0.464 \| 7 \| 0.459 \| \| HG \| 10 \| 0.805 \| 9 \| 0.785 \| 2 \| 0.756 \| 8 \| 0.744 \| \| HGSD \| 10 \| 0.787 \| 9 \| 0.768 \| 2 \| 0.738 \| 8 \| 0.726 \| \| ASW \| 2 \| 0.427 \| 3 \| 0.248 \| 4 \| 0.206 \| 6 \| 0.196 \| \| ASWw \| 2 \| 0.431 \| 3 \| 0.254 \| 4 \| 0.213 \| 10 \| 0.208 \| \| CH \| 2 \| 119.125 \| 3 \| 80.207 \| 4 \| 67.627 \| 5 \| 61.198 \| \| R2 \| 10 \| 0.502 \| 9 \| 0.489 \| 8 \| 0.473 \| 7 \| 0.456 \| \| CHsq \| 2 \| 269.578 \| 3 \| 179.520 \| 4 \| 141.874 \| 5 \| 139.167 \| \| R2sq \| 10 \| 0.720 \| 9 \| 0.704 \| 8 \| 0.689 \| 7 \| 0.666 \| \| HC \| 10 \| 0.102 \| 9 \| 0.111 \| 2 \| 0.127 \| 8 \| 0.131 \| |
| --- | --- | --- | --- | --- | --- | --- | --- | --- | --- | --- | --- | --- | --- | --- | --- | --- | --- | --- | --- | --- | --- | --- | --- | --- | --- | --- | --- | --- | --- | --- | --- | --- | --- | --- | --- | --- | --- | --- | --- | --- | --- | --- | --- | --- | --- | --- | --- | --- | --- | --- | --- | --- | --- | --- | --- | --- | --- | --- | --- | --- | --- | --- | --- | --- | --- | --- | --- | --- | --- | --- | --- | --- | --- | --- | --- | --- | --- | --- | --- | --- | --- | --- | --- | --- | --- | --- | --- | --- | --- | --- | --- | --- | --- | --- | --- | --- | --- | --- | --- | --- | --- | --- | --- | --- | --- | --- | --- | --- | --- | --- | --- | --- | --- | --- | --- | --- | --- | --- | --- | --- | --- | --- | --- | --- | --- | --- | --- | --- | --- | --- | --- | --- | --- | --- | --- | --- | --- | --- | --- | --- | --- | --- | --- | --- | --- | --- | --- | --- | --- | --- | --- | --- | --- | --- | --- | --- | --- | --- | --- | --- | --- | --- | --- | --- | --- | --- | --- | --- | --- | --- | --- | --- | --- | --- | --- | --- | --- | --- | --- | --- | --- | --- | --- | --- | --- | --- | --- | --- | --- | --- | --- | --- | --- | --- | --- | --- | --- | --- | --- | --- | --- | --- | --- | --- | --- | --- | --- | --- | --- | --- | --- | --- | --- | --- | --- | --- | --- | --- | --- | --- | --- | --- | --- | --- | --- | --- | --- | --- | --- | --- | --- | --- | --- | --- | --- | --- | --- | --- | --- | --- | --- | --- | --- | --- | --- | --- | --- | --- | --- | --- | --- | --- | --- | --- | --- | --- | --- | --- | --- | --- | --- | --- | --- | --- | --- | --- | --- | --- | --- | --- | --- | --- | --- | --- | --- | --- | --- | --- | --- | --- | --- | --- | --- | --- | --- | --- | --- | --- | --- | --- | --- | --- | --- | --- | --- | --- | --- | --- | --- | --- | --- | --- | --- | --- | --- | --- | --- | --- | --- | --- | --- | --- | --- | --- | --- | --- | --- | --- | --- | --- | --- | --- | --- | --- | --- | --- | --- | --- | --- | --- | --- | --- | --- |
| *Source: Swiss Household Panel data, 1999–2023* |

| **Table A.2: Multinomial logistic regression for the 1939-1953 cohorts across the three clusters by gender, migration background, and social class. Reference cluster: 1.** | | | | | |
| --- | --- | --- | --- | --- | --- |
| *Cohorts 1939-1953* | Cluster 2 | Cluster 3 |  |  |  |
| Constant | 0.112 (0.134) | -2.714*** (0.289) |  |  |  |
| Man | 1.132*** (0.160) | 2.022*** (0.246) |  |  |  |
| Migration background | -0.842*** (0.255) | 0.887*** (0.280) |  |  |  |
| Erikson-Goldthorpe-Portocarero (EGP) class scheme (Ref.: V Working class (skilled and unskilled)) |  |  |  |  |  |
| IV Petite bourgeoisie (self-employed) | -0.343* (0.184) | -0.044 (0.332) |  |  |  |
| III Routine non-manual employees | -0.198 (0.286) | 0.649 (0.435) |  |  |  |
| II Lower service class | -0.196 (0.263) | 1.143*** (0.358) |  |  |  |
| I Upper service class | -0.639** (0.281) | 1.781*** (0.332) |  |  |  |
| *Note: * p < 0.1, ** p < 0.05, *** p < 0.01, standard errors in parentheses, data: Swiss Household Panel data, 1999–2023, McFadden's Pseudo-R² = 0.116* | | | | | |
|  |  |  |  |  |  |
|  |  |  |  |  |  |
| **Table A.3: Multinomial logistic regression for the 1939-1953 cohorts across the six clusters by gender, migration background, and social class. Reference cluster: 1.** | | | | | |
|  |  | | | | |
| *Cohorts 1954-1968* | Cluster 2 | Cluster 3 | Cluster 4 | Cluster 5 | Cluster 6 |
| Constant | 0.783*** (0.150) | -0.639*** (0.193) | -1.184*** (0.250) | -3.557*** (0.461) | -1.570*** (0.243) |
| Man | 0.933*** (0.201) | 1.849*** (0.223) | -0.051 (0.300) | 2.207*** (0.369) | 1.400*** (0.243) |
| Migration background | -1.302*** (0.264) | -0.644** (0.280) | -0.162 (0.329) | 0.740* (0.387) | 0.386 (0.274) |
| Erikson-Goldthorpe-Portocarero (EGP) class scheme (Ref.: V Working class (skilled and unskilled)) |  | | | | |
| IV Petite bourgeoisie (self-employed) | -0.127 (0.219) | -0.041 (0.259) | 0.420 (0.344) | -0.287 (0.629) | -0.108 (0.347) |
| III Routine non-manual employees | -0.190 (0.291) | 0.214 (0.332) | 0.795** (0.403) | 1.923*** (0.504) | 1.242*** (0.353) |
| II Lower service class | -0.001 (0.345) | 0.953*** (0.359) | 1.671*** (0.412) | 1.698*** (0.588) | 1.757*** (0.388) |
| I Upper service class | 0.190 (0.350) | 0.862** (0.379) | 1.696*** (0.421) | 2.668*** (0.532) | 2.654*** (0.374) |
| *Note: * p < 0.1, ** p < 0.05, *** p < 0.01, standard errors in parentheses, data: Swiss Household Panel data, 1999–2023, McFadden's Pseudo-R² = 0.089* | | | | | |
|  |  |  |  |  |  |
|  |  |  |  |  |  |
| **Table A.4: Multinomial logistic regression for the 1939-1953 cohorts across the three clusters by gender, migration background, and social class. Reference cluster: 1.** | | | | | |
| *Cohorts 1969-1983* | Cluster 2 | Cluster 3 |  |  |  |
| Constant | -0.976*** (0.255) | -1.321** (0.290) |  |  |  |
| Man | 1.232*** (0.293) | -0.025 (0.300) |  |  |  |
| Migration background | -0.877 (0.604) | 0.356 (0.469) |  |  |  |
| Erikson-Goldthorpe-Portocarero (EGP) class scheme (Ref.: V Working class (skilled and unskilled)) |  | |  |  |  |
| IV Petite bourgeoisie (self-employed) | -0.430 (0.474) | 0.851* (0.461) |  |  |  |
| III Routine non-manual employees | 0.090 (0.381) | 0.806* (0.418) |  |  |  |
| II Lower service class | 0.669 (0.445) | 1.827*** (0.461) |  |  |  |
| I Upper service class | -0.197 (0.513) | 1.868*** (0.444) |  |  |  |
| *Note: * p < 0.1, ** p < 0.05, *** p < 0.01, standard errors in parentheses, data: Swiss Household Panel data, 1999–2023, McFadden's Pseudo-R² = 0.087* | | | | | |

| **Table A.5: Multinomial logistic regression for the 1939-1953 cohorts across the three clusters by gender, migration background, and parents’ education. Reference cluster: 1.** | | | | | |
| --- | --- | --- | --- | --- | --- |
| *Cohorts 1939-1953* | Cluster 2 | Cluster 3 |  |  |  |
| Constant | -0.321* (0.181) | -3.305*** (0.392) |  |  |  |
| Man | 1.188*** (0.159) | 2.067*** (0.252) |  |  |  |
| Migration background | -0.582** (0.246) | 0.892*** (0.287) |  |  |  |
| Parents education  (Ref.: Compulsory school) |  |  |  |  |  |
| Vocational education and training | 0.374* (0.195) | 0.631 (0.384) |  |  |  |
| General secondary education | 0.227 (0.306) | 2.150*** (0.442) |  |  |  |
| Higher vocational education  (tertiary VET, tech./voc. schools) | 0.233 (0.268) | 1.332*** (0.431) |  |  |  |
| Tertiary education | -1.107** (0.446) | 2.604*** (0.435) |  |  |  |
| *Note: * p < 0.1, ** p < 0.05, *** p < 0.01, standard errors in parentheses, data: Swiss Household Panel data, 1999–2023, McFadden's Pseudo-R² = 0.140* | | | | | |
|  |  |  |  |  |  |
|  |  |  |  |  |  |
| **Table A.6: Multinomial logistic regression for the 1939-1953 cohorts across the six clusters by gender, migration background, and parents’ education. Reference cluster: 1.** | | | | | |
|  |  | | | | |
| *Cohorts 1954-1968* | Cluster 2 | Cluster 3 | Cluster 4 | Cluster 5 | Cluster 6 |
| Constant | -0.531** (0.208) | -1.104*** (0.271) | -1.491*** (0.364) | -3.987*** (0.621) | -2.794*** (0.445) |
| Man | 0.88*** (0.199) | 1.799*** (0.222) | -0.105 (0.295) | 2.055*** (0.369) | 1.349*** (0.245) |
| Migration background | -1.255*** (0.270) | -0.524* (0.286) | -0.085 (0.335) | 0.701* (0.393) | 0.432 (0.290) |
| Parents education  (Ref.: Compulsory school) |  | | | | |
| Vocational education and training | 0.245 (0.224) | 0.551** (0.277) | 0.774** (0.386) | 1.094* (0.601) | 1.679*** (0.448) |
| General secondary education | 0.789* (0.418) | 1.473*** (0.459) | 2.227*** (0.53) | 2.583*** (0.744) | 3.334*** (0.566) |
| Higher vocational education  (tertiary VET, tech./voc. schools) | 0.712* (0.366) | 1.555*** (0.407) | 1.792*** (0.503) | 2.359*** (0.723) | 2.916*** (0.542) |
| Tertiary education | -0.975 (0.675) | 1.763*** (0.537) | 2.366*** (0.596) | 3.765*** (0.754) | 4.571*** (0.605) |
| *Note: * p < 0.1, ** p < 0.05, *** p < 0.01, standard errors in parentheses, data: Swiss Household Panel data, 1999–2023, McFadden's Pseudo-R² = 0.101* | | | | | |
|  |  |  |  |  |  |
|  |  |  |  |  |  |

| **Table A.7: Multinomial logistic regression for the 1939-1953 cohorts across the three clusters by gender, migration background, and parents’ education. Reference cluster: 1.** | | | | | |
| --- | --- | --- | --- | --- | --- |
| *Cohorts 1969-1983* | Cluster 2 | Cluster 3 |  |  |  |
| Constant | -1.654*** (0.500) | -1.367*** (0.482) |  |  |  |
| Man | 1.1494*** (0.286) | 0.000 (0.307) |  |  |  |
| Migration background | -1.282* (0.661) | 0.316 (0.462) |  |  |  |
| Parents education  (Ref.: Compulsory school) |  | |  |  |  |
| Vocational education and training | 0.812 (0.508) | 0.271 (0.509) |  |  |  |
| General secondary education | 0.783 (0.690) | 1.258** (0.641) |  |  |  |
| Higher vocational education  (tertiary VET, tech./voc. schools) | 1.096* (0.636) | 1.500** (0.612) |  |  |  |
| Tertiary education | 0.965 (0.698) | 2.818*** (0.613) |  |  |  |
| *Note: * p < 0.1, ** p < 0.05, *** p < 0.01, standard errors in parentheses, data: Swiss Household Panel data, 1999–2023, McFadden's Pseudo-R² = 0.120* | | | | | |
